# Modulation of Metabolic Detoxification Pathways Using Foods and Food-Derived Components: A Scientific Review with Clinical Application

**DOI:** 10.1155/2015/760689

**Published:** 2015-06-16

**Authors:** Romilly E. Hodges, Deanna M. Minich

**Affiliations:** ^1^University of Bridgeport, 126 Park Avenue, Bridgeport, CT 07748, USA; ^2^Institute for Functional Medicine, 505 S. 336th Street, Suite 500, Federal Way, WA 98003, USA; ^3^University of Western States, 2900 NE 132nd Avenue, Portland, OR 97230, USA

## Abstract

Research into human biotransformation and elimination systems continues to evolve. Various clinical and *in vivo* studies have been undertaken to evaluate the effects of foods and food-derived components on the activity of detoxification pathways, including phase I cytochrome P450 enzymes, phase II conjugation enzymes, Nrf2 signaling, and metallothionein. This review summarizes the research in this area to date, highlighting the potential for foods and nutrients to support and/or modulate detoxification functions. Clinical applications to alter detoxification pathway activity and improve patient outcomes are considered, drawing on the growing understanding of the relationship between detoxification functions and different disease states, genetic polymorphisms, and drug-nutrient interactions. Some caution is recommended, however, due to the limitations of current research as well as indications that many nutrients exert biphasic, dose-dependent effects and that genetic polymorphisms may alter outcomes. A whole-foods approach may, therefore, be prudent.

## 1. Introduction

Food-based nutrients have been and continue to be investigated for their role in the modulation of metabolic pathways involved in detoxification processes. Several publications to date have leveraged cell, animal, and clinical studies to demonstrate that food-derived components and nutrients can modulate processes of conversion and eventual excretion of toxins from the body [1]. In general, the nature of these findings indicates that specific foods may upregulate or favorably balance metabolic pathways to assist with toxin biotransformation and subsequent elimination [2, 3]. Various whole foods such as cruciferous vegetables [2, 4, 5], berries [6], soy [7], garlic [8, 9], and even spices like turmeric [10, 11] have been suggested to be beneficial and commonly prescribed as part of naturopathic-oriented and functional medicine-based therapies [12, 13].

While these foods are important to note, the science in this active area of inquiry continues to evolve to reveal new findings about food-based nutrients and their effect on health. Thus, the purpose of this review article is to summarize the science to date on the influence of whole foods, with a special focus directed towards phytonutrients and other food-based components, on influencing specific metabolic detoxification pathways, including phase I cytochrome enzymes, phase II conjugation enzymes, antioxidant support systems, and metallothionein upregulation for heavy metal metabolism. Based on this current science, the paper will conclude with clinical recommendations that may be applied in a personalized manner for patients via the discretion of a qualified health professional.

## 2. The Metabolic Pathways of Detoxification

Discussion of physiological pathways for detoxification has been mainly centered around phase I and phase II enzyme systems. This review will cover phase I cytochrome P450 enzymes as well as phase II enzymes, specifically UDP-glucuronosyl transferases, glutathione S-transferases, amino acid transferases, N-acetyl transferases, and methyltransferases. Note that there are other important classes of phase I enzymes, namely, hydroxylation and reduction, which are not covered in this review. While these important enzymes are pivotal to consider, this review of the effect of food on detoxification will also extend into other pathways, including ways to promote gene expression of antioxidant-related enzymes and of metallothionein, an endogenous protein carrier for heavy metals. Each of these four classes of detoxification-related pathways will be discussed within the context of nutrients.

### 2.1. Phase I Cytochrome P450 Enzymes

Initially, the “phases” of detoxification were described as functionalization (or phase I), or the addition of oxygen to form a reactive site on the toxic compound, and conjugation (phase II), or the process of adding a water-soluble group to this now reactive site [14, 15]. The “Phase I” cytochrome P450 superfamily of enzymes (CYP450) is generally the first defense employed by the body to biotransform xenobiotics, steroid hormones, and pharmaceuticals. These microsomal membrane-bound, heme-thiolate proteins, located mainly in the liver, but also in enterocytes, kidneys, lung, and even the brain, are responsible for the oxidation, peroxidation, and reduction of several endogenous and exogenous substrates [13, 15, 16]. Specifically, the function of CYP450 enzymes is to add a reactive group such as a hydroxyl, carboxyl, or an amino group through oxidation, reduction, and/or hydrolysis reactions [15]. These initial reactions have the potential to create oxidative damage within cell systems because of the resulting formation of reactive electrophilic species.

It is accepted that any variability in the number of CYP450 enzymes could have benefit(s) and/or consequence(s) for how an individual responds to the effect(s) of (a) toxin(s). Clinical application of the knowledge of these phase I CYP450 enzymes has been primarily addressed within pharmacology to understand the nature of drug interactions, side effects, and interindividual variability in drug metabolism [15]. The ability of an individual to metabolize 90% of currently used drugs will largely depend on the genetic expression of these enzymes [17]. It is established that many of these CYP450 genes are subject to genetic polymorphisms, resulting in an altered expression and function of individual enzymes. Currently, there exist some laboratory tests to identify the presence of these genetic variants. It is conceivable that having knowledge about foods and their individual (phyto)nutrients, especially in the case of dietary supplements and functional foods, could be worthwhile for clinicians to consider for patients who are taking a polypharmacy approach. Furthermore, as nutritional strategies become more personalized, it would seem that this information could be interfaced with a patient's known CYP450 polymorphisms to determine how to best optimize health outcomes.

#### 2.1.1. CYP1 Enzymes

The CYP1A family is involved in metabolizing procarcinogens, hormones, and pharmaceuticals. It is well-known for its role in the carcinogenic bioactivation of polycyclic aromatic hydrocarbons (PAHs), heterocyclic aromatic amines/amides, polychlorinated biphenyls (PCBs), and other environmental toxins [18, 19]. Low CYP1A2 activity, for example, has been linked to higher risk of testicular cancer [20]. However, due to their rapid conversion to highly reactive intermediates, excessive activity of CYP1A enzymes without adequate phase II support may enhance the destructive effects of environmental procarcinogens [21]. Indeed, genetic polymorphisms in this cytochrome family have been suggested as useful markers for predisposition to certain cancers [15]. CYP1 enzymes are also involved in the formation of clinically relevant estrogen metabolites: CYP1A1/1A2 and CYP1B1 catalyze the 2-hydroxylation and 4-hydroxylation of estrogens, respectively [22]. The potential role of 4-hydroxyestradiol in estrogen-related carcinogenesis, via the production of free radicals and related cellular damage [22], has prompted investigation into factors that modulate CYP1 enzymes.

Various foods and phytonutrients alter CYP1 activity (Tables 1(a) and 1(b)). Cruciferous vegetables have been shown, in humans, to act as inducers of CYP1A1 and 1A2, and animal studies also suggest an upregulation of CYP1B1 [4, 23–27]. The inductory effect of crucifers on CYP1A2 seems especially well established. Clinical studies also indicate that resveratrol and resveratrol-containing foods are CYP1A1 enhancers [28]. Conversely, berries and their constituent polyphenol, ellagic acid, may reduce CYP1A1 overactivity [6], and apiaceous vegetables and quercetin may attenuate excessive CYP1A2 action [24, 29]. Cruciferous vegetables and berries have been suggested as possible modulators of estrogen metabolites: berries for their reducing effect on CYP1A1 [6] and cruciferous vegetables for their stronger induction of CYP1A versus 1B1 enzymes [25–27, 30]. Chrysoeriol, present in rooibos tea and celery, acts selectively to inhibit CYP1B1* in vitro *[31] and may be especially relevant to patients with CYP1B1 overactivity. However, further research is needed to confirm this finding.

Many foods appear to act as both inducers and inhibitors of CYP1 enzymes, an effect which may be dose dependent or altered by the isolation of bioactive compounds derived from food. Curcumin at 0.1% of the diet has been shown, in animals, to induce CYP1A1, for example, [35], yet a diet of 1% turmeric was inhibitory [46]. Black tea at 54 mL/d induced both CYP1A1 and 1A2 [33], yet 20 mg/kg of theaflavins was inhibitory to CYP1A1 [45]. Soybean intake at 100 mg/kg upregulated CYP1A1 activity [7], yet at 1 g/kg black soybean extract [44] and 200 mg daidzein twice daily [49], its effect was inhibitory. Further research is needed to confirm different dose effects and impact in humans.

Varied effects may also occur from different members of the same food group. Seemingly contradictory to research showing that cruciferous vegetables activate CYP1 enzymes, kale (another member of the cruciferous family) appears to inhibit CYP1A2 (as well as 2C19, 2D6, and 3A4) in animals [51]. The dose used, at 2 g/kg per day, is 15-fold higher than the typical level of human consumption [51], and more research would be required to determine whether lower intake levels would also have a similar effect. The same authors also tested the effects of an equivalent volume of cabbage consumption and found no such inhibitory effect, pointing to the possibility that different cruciferous vegetables may have distinct effects on cytochrome activity.

#### 2.1.2. CYP2A-E Enzymes

The large CYP2 family of enzymes is involved in the metabolism of drugs, xenobiotics, hormones, and other endogenous compounds such as ketones, glycerol, and fatty acids [15, 54]. Some notable polymorphisms occur in the CYP2C and CYP2D subgroups, leading to the classification of patients as “poor metabolizers” of various pharmaceuticals: warfarin and CYP2C9, antiarrhythmia agents, metoprolol and propafenone, and CYP2D6, phenytoin, cyclobarbital, omeprazole, and CYP2C19, for example, [15, 17]. CYP2D polymorphisms may be associated with Parkinson's disease and lung cancer [15]. Clinical evidence exists for the induction of CYP2A6 by quercetin and broccoli [4, 29] (Table 2(a)). In animals, chicory appears to induce CYP2A enzymes [41] and rosemary and garlic may upregulate CYP2B activity [9, 37]. Clinical studies using resveratrol and garden cress indicate CYP2D6 inhibition [28, 55] (Table 2(b)). Ellagic acid, green tea, black tea, and cruciferous vegetables also appear to inhibit various CYP2 enzymes.

CYP2E1 enzymes have also attracted particular interest for their role in various diseases. 2E1 metabolizes nervous system agents such as halothane, isoflurane, chlorzoxazone, and ethanol and bioactivates procarcinogenic nitrosamines and aflatoxin B1 [15, 65]. It produces free radicals regardless of substrate [15], and CYP2E1 polymorphisms have been associated with altered risk for coronary artery disease [66] and gastric cancer [67]. CYP2E1-induced oxidative stress has also been shown to lead to impaired insulin action via the suppression of GLUT4 expression [68]. Attenuation of 2E1 overactivity may therefore be an important consideration in high-risk patients.

Watercress and garlic are CYP2E1 inhibitors in humans [59, 60].* In vivo* evidence also suggests that N-acetyl cysteine, ellagic acid, green tea, black tea, dandelion, chrysin, and medium chain triglycerides (MCTs) may downregulate CYP2E1 [33, 43, 54, 61, 63, 64]. MCT oil may specifically attenuate the ethanol-induced upregulation of CYP2E1 and production of mitochondrial 4-hydroxynonenal, a marker of oxidative stress [64].

#### 2.1.3. CYP3A Enzymes

The occurrence of the different CYP3A isoforms is tissue-specific [15]. Rooibos tea, garlic, and fish oil appear to induce the activity of CYP3A, 3A1, and 3A2 [8, 36, 69, 70] (Table 3(a)). Possible inhibitory foods include green tea, black tea, and quercetin [33, 56, 71, 72] (Table 3(b)). The most clinically relevant of the enzymes is CYP3A4, which is expressed mainly in the liver and to a lesser extent in the kidney [13]. Caffeine, testosterone, progesterone, and androstenedione are substrates of the CYP3A4 enzyme system, as are various procarcinogens including PAHs and aflatoxin B1 [15]. To date, however, the principal driver for research on CYP3A4 has been due to its role in the metabolism of over 50 percent of all pharmaceuticals [73]. The potential for drug interaction with this single enzyme, coupled with the wide interindividual differences in enzymatic activity, generates some level of risk in administration of high doses and multiple drugs as well as food-drug and herb-drug interactions. Grapefruit juice is perhaps the most well-known food inhibitor of this enzyme [74], though resveratrol and garden cress, a member of the cruciferous vegetable family, appear to have similar effects in humans, albeit at intakes above what would be expected without high-dose supplementation [28, 55]. Curcumin may upregulate 3A4 activity [11].

Once again, there are indications that a biphasic effect may be seen from dietary bioactive compounds; Davenport and Wargovich (2005) found that shorter-term or lower dosing with garlic organosulfur compounds produced potentially anticarcinogenic effects but that longer-term higher doses (200 mg/kg) of allyl sulfides led to minor hepatic toxicity [8]. One garlic clove contains only 2,500–4,500 *μ*g of the allyl sulfide precursor, allicin [76], so the higher dose is much more than would be consumed in a typical human diet. In another example, two components of cruciferous vegetables, sulforaphanes and indole-3-carbinol, inhibited and increased activity, respectively [57, 75], highlighting the potential for human studies using whole foods to clarify the outcome of consumption.

#### 2.1.4. CYP4 Enzymes

Less is known about this family of enzymes, since it is thought to play a smaller role in drug metabolism. It is, however, understood to be a primarily extrahepatic family of cytochromes, inducible by clofibrate and ciprofibrate (hypolipidemic drugs), NSAIDs, prostaglandins, and toxicants such as phthalate esters [15, 77]. The CYP4B1 isoform is involved in the metabolism of MCTs (medium chain triglycerides), as well as the bioactivation of pneumotoxic and carcinogenic compounds [78].

Polymorphisms and overexpression of this subgroup may be associated with bladder cancer [15] and colitis [79]. A report by Ye et al. (2009) which examined the link between colitis and CYP4B1 activity found that the promotion of CYP4B1 activity by caffeic acid (found in caffeine-containing foods) (Table 4) correlated with reduced inflammation and disease activity [79]. Green tea may act to induce CYP4A1, as suggested by animal studies [40]. More research is needed to clearly identify food influences on this enzyme family.

### 2.2. Phase II Conjugation Enzymes

After a xenobiotic has gone through the process of becoming hydrophilic through reactions overseen by CYP450 enzymes, its reactive site can be conjugated with an endogenous hydrophilic substance. This reaction is often referred to as “phase II detoxification.” Conjugation involves the transfer of a number of hydrophilic compounds (via their corresponding enzymes), including glucuronic acid (glucuronyl transferases), sulfate (sulfotransferases), glutathione (glutathione transferases), amino acids (amino acid transferases), an acetyl group (N-acetyl transferases), and a methyl group (N- and O-methyltransferases) [81]. The result of the collective activity of these enzymes is an increase in the hydrophilicity of the metabolite, theoretically leading to enhanced excretion in the bile and/or urine [81]. Similar to the CYP450 enzymes, genetic polymorphisms can have profound influence on the function of these conjugating enzymes [82], with potential implication in the development of several forms of cancer [83].

It is conceivable that modulation of phase II enzymes by food-based bioactive compounds may be advantageous in patients who have altered enzyme activity due to genetic polymorphisms or who have a high toxic burden due to chronic exposure to environmental pollutants, overactive phase I activity, or hormonal imbalance. For example, James et al. (2008) suggest that upregulation of glucuronidation and sulfonation by certain bioactive compounds may be a useful consideration for the elimination of environmental PCBs [19].

#### 2.2.1. UDP-Glucuronosyltransferases

This class of enzymes, comprising multiple proteins and even subfamilies, plays an essential role in enhancing the elimination of biotransformed toxins in urine and feces, as well as metabolizing steroid hormones and bilirubin [84, 85]. Their function is to catalyze the covalent linkage of glucuronic acid from UDP-glucuronic acid to an accepting functional group on the molecule, a process referred to as glucuronidation [86]. Glucuronidation occurs primarily in the liver but can occur in other tissues, such as the small intestine [86, 87]. Bilirubin, specifically, is principally conjugated by UGT1A1 in hepatocytes [88] and then excreted with bile into the intestinal tract. It has been estimated that 40–70% of all medications are subject to glucuronidation reactions in humans, thereby suggesting the significance of this conjugation enzyme family [88]. Since UDP-glucuronosyltransferases (UGTs) also metabolize phytochemicals, alterations in their effects may be seen with genetically downregulated enzyme activity; flavonoids are conjugated with glucuronide and sulfate; therefore, UGT or sulfotransferase (SULT) polymorphisms may produce variability in phytochemical clearance and efficacy [89].

Clinical and observational studies point to cruciferous vegetables, resveratrol, and citrus as foods and bioactive compounds that induce UGT enzymes [25, 28, 90–92] (Table 5(a)). Animal studies also suggest the potential for other foods and nutrients, including dandelion, rooibos tea, honeybush tea, rosemary, soy, ellagic acid, ferulic acid, curcumin, and astaxanthin, to enhance UGT activity [37, 39, 53, 93–95]. Interestingly, the effect of resveratrol was seen only in individuals with low baseline enzyme levels/activity, suggesting that some phytochemicals may modulate, rather than outright induce, enzymatic activity [28]. In addition, many studies note that effects are variable depending on gender and genotype [85, 90, 92]; for example, women with the UGT1A1 *∗*28 polymorphism (7/7) were responsive to citrus intervention, whereas those with other genetic variants were not [92].

Meaningful interpretations of these studies may still be elusive, however: in one combined dietary trial, the consumption of 10 servings per day of a combination of cruciferous vegetables, soy foods, and citrus fruits did not have a significant effect on UGT enzyme activity compared with a diet devoid of fruits and vegetables [85]. The authors hypothesize that these results may be due to their choice of specific foods within those groups or due to Nrf2 activation (discussed in subsequent sections) when fruits and vegetables were avoided.

The effects of UGT activity may also be enhanced by D-glucaric acid by theoretical inhibition of beta-glucuronidase enzymes [100]. Beta-glucuronidase enzymes act to reverse UGT conjugation reactions. D-glucaric acid is found in many fruits, vegetables and legumes (Table 5(b)). When tested in humans, however, a diet supplemented with cruciferous vegetables (2/3 cup broccoli, 1/2 cup cabbage, and 1/2 cup radish sprouts), citrus fruits (1 cup grapefruit juice, 1/2 cup orange juice, 1 cup orange/grapefruit segments, and 1 orange peel), and soy foods was found to have no effect on beta-glucuronidase activity [101] (amounts standardized for 55 kg body weight), indicating that the clinical effects of D-glucaric acid consumption still need further clarification.

*In vivo* research suggests that polyphenol extracts of certain berries, specifically strawberries and blackcurrant, may inhibit beta-glucuronidase activity in the intestinal lumen; Kosmala et al. (2014) observed this effect using both strawberry pomace water extract and water-alcohol extract containing 5.1% and 17.1% ellagic acid, and 0.2% and 10.9% proanthocyanidins, respectively [100]. Jurgoński et al. (2014) found a similar inhibitory effect using a diet of 1.5% blackcurrant extract (total polyphenolic content 66.8 g/100 g extract) [102]. Interestingly, the highest levels of beta-glucuronidase activity were seen in rabbits fed a high fat diet (32% calories from fat, including 10% from lard), without blackcurrant extract supplementation, suggesting that dietary fat may also alter enzyme activity [102].

Inhibition of UGT enzymatic activity may be a consideration for modulation of hormone levels and the risk of certain cancers, such as prostate cancer [84].* In vitro* studies suggest that various foods and food-based components may inhibit UGT activity, including green and black tea, quercetin, rutin, naringenin, allspice, peppermint oil, cacao, and silymarin [84], although further research is needed to evaluate their* in vivo* and clinical effects.

#### 2.2.2. Sulfotransferases

As the name of this superfamily of enzymes might suggest, SULTs are responsible for the transfer of a sulfuryl group donated by 3′-phosphoadenosine-5′-phosphosulfate (PAPS) to hydroxyl or amine groups, particularly in the areas of liver, intestine, adrenal gland, brain, and skin tissues [103]. This process is often referred to as sulfation but is more accurately termed sulfonation or sulfurylation. Decreased function of these enzymes, through genetic variability or presence of environmental chemicals, can lead to eventual interference with thyroid hormone, estrogen, and androgen levels [104, 105], as well as variable polyphenol effects [106], since the active forms of these compounds can be degraded via sulfonation. Typically, once compounds have been conjugated with sulfate, there is less reactivity and toxicity incurred from the precursor molecule [105].

Few* in vivo *studies have examined the effects of dietary components on SULT activity, although caffeine and retinoic acid are possible SULT inducers according to animal studies [107, 108] (Table 6(a)). Although it is uncertain how their outcomes will translate* in vivo*, various* in vitro* studies have indicated the possibility of sulfotransferase inhibition (including competitive inhibition) by wine anthocyanins and flavonols, synthetic food colors (especially red colors), apple and grape juice, catechins including epigallocatechin gallate, quercetin, curcumin, resveratrol, flavonoids (apigenin, chrysin, fisetin, galangin, kaempferol, quercetin, myricetin, naringenin, and naringin), and certain phytoestrogens (daidzein, genistein) [3, 105]. Pyridoxal-6-phosphate, the active form of vitamin B6 (which is widely distributed in foods), may also be a competitive SULT inhibitor, according to one* in vitro *study [109], although human tissue concentrations and clinical effects may be vastly different. Of note, caffeic acid demonstrates* in vitro *SULT-inhibitory properties [105]. This finding conflicts with its* in vivo* ability to induce SULT enzymes, as described by Zhou et al. (2012) [107], highlighting the difficulty of extrapolating meaningful conclusions from* in vitro* data.

SULT enzyme activity is dependent on a depletable reserve of inorganic sulfate [112]. Dietary sources of sulfur-containing compounds may therefore play an essential role in SULT function, by providing the substrate for enzyme action (Table 6(b)).

#### 2.2.3. Glutathione S-Transferases

Similar to the aforementioned categories of conjugating enzymes, glutathione S-transferases (GSTs) include a complex of enzymes, whose main function is to attach a glutathione group to a biotransformed metabolite. The production of these enzymes can be induced through the production of reactive oxygen species and via gene transcription involving the antioxidant-responsive element (ARE) and the xenobiotic-responsive element (XRE), which will be subsequently discussed in this paper [113].

Cruciferous and allium vegetables and resveratrol demonstrate ability to induce GSTs in humans [28, 114–117] (Table 7(a)). Observational research also associates citrus consumption with increased GST activity [115].* In vivo *data also suggest many foods and food constituents to be upregulators of these enzymes, including garlic, fish oil, black soybean, purple sweet potato, curcumin, green tea, rooibos tea, honeybush tea, ellagic acid, rosemary, ghee, and genistein [36, 43, 44, 70, 93, 118–123]. Conjugated linoleic acid has been shown to be at least partly responsible for the effect of ghee [122]. It is possible that the effects of at least some of these foods and bioactive compounds may be due to their upregulation of the Nrf2 signaling pathway.

Genetic variances, gender, and even possibly body weight appear to play a role in the effects of dietary factors on GST enzymes [114–116]. Clinical investigation of cruciferous and allium vegetables by Lampe et al. (2000) found that an upregulated effect was most marked in women, indicating gender variability, and that the effect was also genotype-dependent, occurring only in GSTM1-null individuals [116]. The same investigators also found that apiaceous vegetables inhibited GST activity, but only in GSTM1+ men [116] (Table 7(b)). High doses of quercetin and genistein have also shown inhibitory effects [123, 126].

There is evidence that at least some of these foods and phytonutrients may exert modulatory rather than absolute inductive/inhibitory effects; Chow et al. (2010) found that resveratrol increased GST only in those with low baseline enzyme levels or activity [28]. It is also noteworthy that bioactive components of crucifers, including isothiocyanates, are substrates for GST enzymes and that GST genotype may therefore alter the response to cruciferous vegetables consumption on other mechanisms such as glutathione peroxidase and superoxide dismutase [134, 135]. GSTM1-null genotype is associated with a more rapid excretion of isothiocyanates, leading some researchers to conclude that the benefits of cruciferous vegetable consumption may be lessened in individuals with this genetic variation [89].

Support for glutathione conjugation also involves enhancing reduced glutathione (GSH) status. Glutathione is a low-molecular weight tripeptide containing residues of cysteine, glutamate, and glycine [136]. Most glutathione from foods and supplements is poorly absorbed, so liposomal delivery has been used [137]. The sulfur-containing amino acids methionine and cystine are important precursors to glutathione formation; their depletion leads to depressed GSH levels [138]. N-acetyl cysteine has also been used to restore depleted GSH levels in a clinical setting [139].

Various nutrients may also enhance endogenous glutathione synthesis, including vitamin B6, magnesium, and selenium [140, 141]. Curcuminoids (from turmeric), silymarin (from milk thistle), folic acid, and alpha-lipoic acid have been shown, in humans, to restore depleted GSH [129, 130, 142, 143]. In animal studies, cruciferous vegetables and artichoke have also demonstrated a GSH-protective effect [131–133]. There is therefore the potential to improve glutathione status via diet or supplementation (Table 7(c)).

#### 2.2.4. Amino Acid Transferases

Amino acids of various types (e.g., taurine, glycine), whether endogenous or exogenous (from dietary sources) in origin, can be utilized for attaching to molecules for their excretion. For the benefit of providing a substrate to these enzymes, it is generally thought that dietary protein is required for an effective detoxification protocol.  Table 8 lists amino acids used in phase II conjugation reactions and selected food sources.

#### 2.2.5. N-Acetyl Transferases (NAT)

This class of enzymes is responsible for the transfer of an acetyl group to convert aromatic amines or hydrazines to aromatic amides and hydrazides, which is significant for those taking pharmaceuticals such as isoniazid, hydralazine, and sulphonamides [83]. Polymorphisms in genes for this category of enzymes, leading to slow metabolism, have been shown to be associated with hepatoxicity during drug treatment [146]. One small human study found that 500 mg quercetin daily enhanced NAT activity [29]. However, more research is needed to understand the relationship between dietary nutrients and NAT function.

#### 2.2.6. Methyltransferases

Relatively significant attention has been given in various medical communities to this class of phase II enzymes due to the increasing importance of methylation for reducing disease risk. The conjugating donor compound in methyltransferase reactions is a methionine group from S-adenosyl-L-methionine (SAMe) [147]. Catechol O-methyltransferase (COMT) is one of the prominent methyltransferases that has received wide attention due to its role in estrogen detoxification [148].

Support for methylation consists of nutrient cofactors and methyl donors, such as methionine, vitamin B12, vitamin B6, betaine, folate, and magnesium [144]. Various foods can provide these nutrients (Table 9). Conversely, a high sucrose diet may inhibit methylation enzymes such as COMT [149].

## 3. Gene Induction of Phase II Detoxification and Antioxidant Enzymes through Nrf2

**Figure 1:**
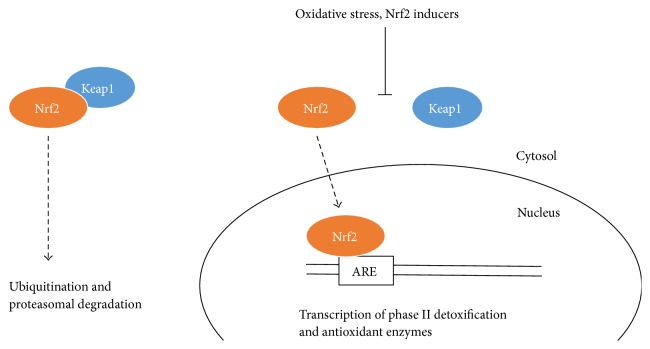
Nrf2/Keap1 signaling (created from text in [154]).

The transcription factor, Nrf2 [nuclear factor erythroid 2 (NF-E2) p45-related factor 2], is key to regulating the body's detoxification and antioxidant system. When activated, Nrf2 dissociates from the cytosolic protein, Keap1 (Kelch-like ECH associated protein 1), and translocates to the nucleus to bind to AREs in the promoter/enhancer portion of genes associated with phase II detoxification and antioxidant enzyme genes [150] (Figure 1). Nrf2-deficient animals experience increased toxicity from drugs [151], carcinogens, allergens, and environmental pollutants [152] and do not respond as well to the anti-inflammatory effects of phytochemicals [153], indicating the essentiality of these enzymes. Conversely, Nrf2 induction is considered protective against various oxidative stress-related conditions such as cancer, kidney dysfunction, pulmonary disorders, arthritis, neurological disease, and cardiovascular disease [154].

Research demonstrates that dietary components, especially phytochemicals, not only scavenge reactive oxygen species, thereby acting as direct antioxidants, but also regulate Nrf2 activity [150].* In vivo *evidence exists for Nrf2-modulation by curcumin [155–158], broccoli constituents [159, 160], garlic [161–163], epicatechins [164–167], resveratrol [168, 169], ginger [170, 171], purple sweet potato [118], isoflavones [172, 173], coffee [174], rosemary [175, 176], blueberry [166, 177], pomegranate [178], naringenin [179], ellagic acid [166], astaxanthin [166], and *γ*-tocopherol [180] (Table 10(a)). A clinical trial by Magbanua et al. (2011), investigating the Nrf2 modulation effects of fish oil and lycopene in the context of prostate cancer risk, also demonstrated that these dietary compounds can upregulate Nrf2 signaling and response to oxidative stress in humans [181]. Direct comparison of the magnitude of effect between these compounds can be difficult to gauge. Some information on their relative effects is provided by Kavitha et al. (2013), who ranked the order of potency of the compounds they tested (from highest to lowest) as chlorophyllin (a semisynthetic compound derived from chlorophyll), blueberry, ellagic acid, astaxanthin, and EGCG [166].

Various studies point to the advantageous effects of whole foods, and food combinations, versus specific bioactive compounds. Zhou et al. (2014), for example, illustrate how organosulfur compounds are not the only Nrf2-enhancing bioactive compounds in garlic; garlic carbohydrate derivatives also show Nrf2-modulatory activity [186]. Balstad et al. (2011), in testing the effects of a combination of food extracts on Nrf2 activity in mice, found that the combination produced a larger-than-expected effect, indicating an additive or synergistic effect [176]. By their calculations, the food extract they used equated to a human (70 kg) dose of 14–23 g each of turmeric, rosemary, and thyme, which is clearly not practical for clinical application, as well as 140–233 g each of coffee, red onion, and broccoli. Calabrese et al. (2010) and Houghton et al. (2013) have also argued that Nrf2 inducers exhibit biphasic effects, with lower doses demonstrating stimulatory effects and higher doses exhibiting Nrf2-interference [187, 188]. These data suggest that the doses found in whole foods may be more beneficial than supplements at supraphysiological doses. In fact, it may well be their weak prooxidant effects that stimulate Nrf2 inducers' favorable antioxidant responses [188].

Nonuniform activities of different foods within the same food group should, once again, be considered; in their recent review of the effects of plant-derived compounds on Nrf2 activation, Stefanson and Bakovic (2014) noted that pak choi, via presumed Nrf2 activation, was more effective at reducing inflammation in the colon than broccoli and that broccoli upregulated some additional Nrf2-related antioxidant enzymes compared with pak choi [189]. Interestingly, this effect was only apparent when steamed, rather than cooked, broccoli was used [189], indicating that food preparation may be an important consideration.

Conversely to its role in cancer prevention, overexpression of Nrf2 is found in many cancer cells and has been shown to promote tumor growth and resistance to anticancer therapy [154]. Consequently, the inhibition of Nrf2 signaling may be clinically relevant for patients receiving cancer chemotherapy [184, 185]. Overexpression of Nrf2 and CYP2E1 has also been associated with impaired GLUT4 activity and insulin resistance [68]. As noted above, supplementation (above levels normally consumed through diet) with certain phytochemicals may have inhibitory effects on Nrf2 activation, including luteolin [184] and quercetin [185] (Table 10(b)). Vitamins A, C, and E and N-acetyl cysteine have also been implicated as Nrf2 inhibitors at high doses [188]. These findings point to the need for further research to clarify outcomes as they relate to specific disease states as well as potential biphasic dose effects.

## 4. Metallothionein

Metallothionein, a cysteine-rich protein with the ability to bind divalent cations, including toxic metals such as mercury, cadmium, lead, and arsenic, is gaining recognition as an important component in heavy metal detoxification [190–192]. Similar to the upregulation of phase II and antioxidant enzymes, metallothionein can be induced at specific promoter regions of genes by stimuli such as heavy metals, oxidative stress, glucocorticoids, and even zinc [192]. In addition to sequestering heavy metals, it is capable of scavenging free radicals and reducing injury from oxidative stress [192], as well as inhibiting NF-*κ*B signaling [193].

Dietary patterns and nutrients may result in changes in metallothionein production. Lamb et al. (2011) reported a 54% increase in metallothionein mRNA production in a small clinical trial in women with fibromyalgia following an elimination diet in conjunction with a phytonutrient-rich medical food consisting of hops, pomegranate, prune skin, and watercress [194]. Zinc supplementation (15 mg/day) to healthy men over 10 days led to significantly increased metallothionein mRNA, up to 2-fold in leukocytes and up to 4-fold from dried blood spots [195]. Metallothionein has been shown to be decreased in the intestinal mucosa of patients with inflammatory bowel disease (IBD); however, zinc supplementation (300 mg zinc aspartate, equal to 60 mg elemental zinc per day for 4 weeks) in 14 zinc-deficient patients with IBD resulted in slightly higher metallothionein concentration in the intestinal mucosa [196]. Cruciferous phytonutrients may also modulate metallothionein expression, as suggested by a 10-fold increase following a single oral dose of 50 *μ*mol sulforaphane to rats [197]. Chromium may* inhibit* zinc-induced metallothionein expression, according to animal studies by Kimura et al. (2011) [198]. Early-stage,* in vitro* studies also suggest that quercetin and* Cordyceps sinensis*, a mushroom native to the Himalayan region, may upregulate metallothionein expression [199, 200].

## 5. Clinical Applications

With the continued emergence of data supporting the role of toxins in chronic disease processes, it is becoming increasingly necessary for clinicians to understand how to provide therapeutic modalities to reduce toxin load in patients. In this paper, several studies regarding the influence of foods and food-based nutrients on the systems of detoxification were presented. From the current information presented, listed below are some key concepts for translation into the clinical setting.

### 5.1. Nonclinical versus Clinical Studies

One of the limitations that comes to the forefront in this collection of studies is how the information, in many cases, is constrained primarily to studies in cells or animals. It remains questionable as to whether similar effects would be seen in humans at moderate, reasonable doses. In the cell studies, it is difficult to anticipate findings due to the lack of pleiotropic activity that occurs in a complex, living system with multiple detoxification systems working simultaneously. Along similar lines, animal studies are often difficult to extrapolate to individuals due to the degree of variability in genotype and environmental phenotype seen in the diverse human population. Therefore, at this time, it is best to take precaution in firmly advocating foods or food-based nutrients that only have cell or animal data as support. It is best to rely on the clinical studies that have been published to date in making more firm recommendations.

### 5.2. Single Agent versus Lifestyle

While this paper focuses on isolated nutrients and foods that contain those nutrients, it might be optimal from a clinical perspective to consider how an entire lifestyle might induce or inhibit the array of detoxification enzymes. For example, this paper has not addressed behaviors like smoking, physical activity, or stress. The modern clinician needs to weigh all these variables against each other. Yet, science has not fully demonstrated the individual impacts of these factors, along with all of them together. Therefore, at this time, a dietary pattern favoring whole, unprocessed, plant-based foods and the removal or reduction of toxic substances in one's environment is a two-prong approach that would seem to have the best overarching scientific underpinning.

### 5.3. Modulating versus Inhibiting/Inducing Effects

In several instances, certain foods exhibited a particular activity on an enzyme, while, at higher doses, they had another, opposite effect. Essentially, many foods serve as what is commonly referred to as being “bifunctional modulators,” possessing the ability to effectively induce or inhibit detoxification enzyme activity based on the dose response. Therefore, the resulting clinical takeaway might be to encourage patients to follow a mixed, varied diet, full of different plant-based, whole foods. Smaller amounts of many compounds might be more therapeutic and supportive for biochemical pathways rather than overriding signals derived from high concentrations of nutrients through high-dose supplementation or the repeat, daily ingestion of large quantities of the same food.

### 5.4. Polypharmacy

For patients who are taking multiple pharmaceuticals, it is important to know which detoxification systems will be influenced by nutrients and foods so that side effects are minimized or avoided.

### 5.5. Dietary Supplements versus Foods

Since there can be potent effects of food-based nutrients on detoxification pathways, it would be best for the average patient to follow, as indicated above, a mixed, complex, and whole-foods diet. Additionally, dietary supplements may be a helpful adjunct in patients in which the practitioner has information about the patient's genetic variability, so that nutrients can be tailored accordingly. Without a full understanding of a patient's SNPs (single nucleotide polymorphisms), it becomes difficult to make accurate assessments about nutrients and dosing.

### 5.6. Duration of Dosing

Another factor to consider in therapeutic intervention is the timing and duration of the dose of nutrient or the food. In some of the research presented here, effects on detoxification enzymes were seen after several days of food intake or supplementation, while, in other cases, induction of an enzyme might be fairly rapid, followed by efficient adaptability. This variable needs to be considered in further clinical research and requires close monitoring in clinical practice.

### 5.7. Foods Known to Impact Detoxification

Based on the four systems examined in this paper, there are several foods which seem to have demonstrated an influence on detoxification systems. Many of them have been acknowledged as part of naturopathic medicine. Hence, it would be useful to have a knowledge base of this cumulative set of foods as patients embark upon detoxification protocols. This recent scientific update notes clinical evidence of effects from cruciferous vegetables (in combination, and specifically watercress, garden cress, and broccoli), allium vegetables, apiaceous vegetables, grapefruit, resveratrol, fish oil, quercetin, daidzein, and lycopene. Many other foods, beverages, and nutrient bioactive compounds, based on this review of scientific literature, are also suggested as modulators of detoxification enzymes* in vivo* (Table 11).

## 6. Conclusions

Over the past decade, there has been investigation into nutrigenomic and epigenetic influences of food constituents on chronic diseases [201, 202]. Similarly, studies have revealed that exposure to and accumulation of toxins play a significant role in cardiovascular disease, type 2 diabetes, and obesity [203–207]. Thus, one's dietary intake and environmental influences may have large bearing on the incidence of chronic disease. In fact, these influences may be significant not just for the individual, but for several generations due to the transgenerational inheritance of epigenetic changes [208, 209]. Therefore, it would seem that designing clinical recommendations to maximize the effects of food and reduce the impact of toxins is essential. However, it is not without caution and critical thinking that a detoxification protocol should be assembled for patients by trained clinicians. There remain many unresolved issues regarding knowing how and what foods modulate detoxification pathways.

## Figures and Tables

**(a):** 

Enzyme	Food, beverage, or bioactive compounds *Food sources in italics *	Type of study	Dosages used and references
	Cruciferous vegetables	Clinical	500 mg/d indole-3-carbinol [23]
	Resveratrol *Grapes, wine, peanuts, soy*, and *itadori tea *[32]	Clinical	1 g/d resveratrol [28]: *note high dose used *
	Green tea	*In vivo *	45 mL/d/rat (avg. 150 g animal weight) green tea [33]
	Black tea	*In vivo *	54 mL/d/rat (avg. 150 g animal weight) black tea [33]
CYP1A1	Curcumin *Turmeric, curry powder* [34]	*In vivo *	1,000 mg/kg/d/rat curcumin [35], or about 150 mg per rat per day
	Soybean	*In vivo *	100 mg/kg soybean extract [7]
	Garlic	*In vivo *	30 to 200 mg/kg garlic oil [36]
	Fish oil	*In vivo *	20.5 g/kg fish oil [36]: *note high dose used *
	Rosemary	*In vivo *	Diet of 0.5% rosemary extract [37]
	Astaxanthin *Algae, yeast, salmon, trout, krill, shrimp*, and* crayfish *[38]	*In vivo *	Diets of 0.001–0.03% astaxanthin for 15 days [39]

	Cruciferous vegetables	Clinical	7–14 g/kg cruciferous vegetables including frozen broccoli and cauliflower, fresh daikon radish sprouts and raw shredded cabbage, and red and green [24] 500 g/d broccoli [4] 250 g/d each of Brussel sprouts and broccoli [25] 500 g/d broccoli [26]
CYP1A2	Green tea	*In vivo *	45 mL/d/rat (avg. 150 g animal weight) green tea [33] Green tea (2.5% w/v) as sole beverage [40]
	Black tea	*In vivo *	54 mL/d/rat (avg. 150 g animal weight) black tea [33]
	Chicory root	*In vivo *	Diet of 10% dried chicory root [41]
	Astaxanthin *Algae, yeast, salmon, trout, krill, shrimp*, and* crayfish* [38]	*In vivo *	Diets of 0.001–0.03% astaxanthin for 15 days [39]

CYP1B1	Curcumin *Turmeric, curry powder* [34]	*In vivo *	Diet of 0.1% curcumin [35]
	Cruciferous vegtables	*In vivo *	25–250 mg/kg indole-3-carbinol [27]

**(b):** 

Enzyme	Food, beverage, or bioactive compounds *Food sources in italics *	Type of study	Dosages used and references
	Black raspberry	*In vivo *	Diet of 2.5% black raspberry [6]
	Blueberry	*In vivo *	Diet of 2.5% blueberry [6]
CYP1A1	Ellagic acid *Berries, pomegranate, grapes, walnuts*, and *blackcurrants* [42]	*In vivo *	30 mg/kg/d ellagic acid [43] 400 ppm ellagic acid [6]
	Black soybean	*In vivo *	1 g/kg black soybean seed coat extract [44]: *note high dose used *
	Black tea	*In vivo *	20 mg/kg theaflavins [45]
	Turmeric	*In vivo *	Diet of 1% turmeric [46]

	Apiaceous vegetables	Clinical	4 g/kg apiaceous vegetables, including frozen carrots and fresh celery, dill, parsley, and parsnips [24]
	Quercetin *Apple, apricot, blueberries, yellow onion, kale, alfalfa sprouts, green beans, broccoli, black tea*, and *chili powder* [47, 48]	Clinical	500 mg/d quercetin [29]
CYP1A2	Daidzein *Soybean* [49]	Clinical	200 mg twice daily dosing of daidzein [49]
	Grapefruit	Clinical	300 mL grapefruit juice [50]
	Kale	*In vivo *	2 g/kg/d kale, as freeze-dried kale drink [51]
	Garlic	*In vivo *	100 mg/kg garlic oil [52]
	Chamomile	*In vivo *	Free access to 2% chamomile tea solution [53]
	Peppermint	*In vivo *	Free access to 2% peppermint tea solution [53]
	Dandelion	*In vivo *	Free access to 2% dandelion tea solution [53]
	Turmeric	*In vivo *	Diet of 1% turmeric [46]

**(a):** 

Enzyme	Food, beverage, or bioactive compounds *Food sources in italics *	Type of study	Dosages used and references
CYP2A	Chicory root	*In vivo *	Diet of 10% dried chicory root [41]

CYP2A6	Quercetin *Apple, apricot, blueberries, yellow onion, kale, alfalfa sprouts, green beans, broccoli, black tea*, and *chili powder* [47, 48]	Clinical	500 mg/d quercetin [29]
	Broccoli	Clinical	500 g/d broccoli [4]

CYP2B1	Rosemary	*In vivo *	Diet of 0.5% rosemary extract [37]
	Garlic	*In vivo *	0.5 and 2.0 mmol/kg diallyl sulfide, or about 75 and 300 mg, respectively [9]

CYP2B2	Rosemary	*In vivo *	Diet of 0.5% rosemary extract [37]

CYP2E1	Fish oil	*In vivo *	20.5 g/kg fish oil [36]: *note high dose used *
	Chicory root	*In vivo *	Diet of 10% dried chicory root [41]

**(b):** 

Enzyme	Food, beverage, or bioactive compounds *Food sources in italics *	Type of study	Dosages used and references
	Ellagic acid *Berries, pomegranate, grapes, walnuts, *and *blackcurrants *[42]	*In vivo *	10 and 30 mg/kg/d ellagic acid [43]
CYP2B	Green tea	*In vivo *	100 mg/kg/d green tea extract [56]
	Cruciferous vegetables	*In vivo *	3 and 12 mg/kg/d sulforaphane [57]

CYP2B1	Turmeric	*In vivo *	Diet of 1% turmeric [46]

	Green tea	*In vivo *	45 mL/d/rat (avg. 150 g animal weight) green tea [33]
CYP2C	Black tea	*In vivo *	54 mL/d/rat (avg. 150 g animal weight) black tea [33]
	Ellagic acid *Berries, pomegranate, grapes, walnuts, *and *blackcurrants* [42]	*In vivo *	30 mg/kg/d ellagic acid [43]

CYP2C6	Ellagic acid *Berries, pomegranate, grapes, walnuts, *and *blackcurrants* [42]	*In vivo *	30 mg/kg/d ellagic acid [43]

CYP2C9	Resveratrol *Grapes, wine, peanuts, soy, *and *itadori tea* [32]	Clinical	1 g/d resveratrol [28]: *note high dose used *
	Myricetin *Onions, berries, grapes,* and* red wine* [58]	*In vivo *	2 and 8 mg/kg myricetin [58]

CYP2C19	Kale	*In vivo *	2 g/kg/d kale, as freeze-dried kale drink [51]

	Resveratrol *Grapes, wine, peanuts, soy, *and *itadori tea* [32]	*Clinical *	1 g/d resveratrol [28]: *note high dose used *
CYP2D6	Garden cress	Clinical	7.5 g twice daily intake of garden cress seed powder [55]
	Kale	*In vivo *	2 g/kg/d kale, as freeze-dried kale drink [51]

	Watercress	Clinical	50 g watercress homogenate [59]
	Garlic	Clinical and *in vivo *	0.2 mg/kg diallyl sulfide, equivalent to high human garlic consumption [60] 100 mg/kg garlic oil [52] 200 mg/kg diallyl sulfide [8] 30 to 200 mg/kg garlic oil [36] Diet of 2% and 5% garlic powder [61]
	N-acetyl cysteine *Allium vegetables* [54]	*In vivo *	25 mg/kg and 50 mg/kg N-acetyl cysteine [54]
CYP2E1	Ellagic acid *Berries, pomegranate, grapes, walnuts, *and *blackcurrants* [42]	*In vivo *	10 and 30 mg/kg/d ellagic acid [43]
	Green tea	*In vivo *	45 mL/d/rat (avg. 150 g animal weight) green tea [33]
	Black tea	*In vivo *	54 mL/d/rat (avg. 150 g animal weight) black tea [33]
	Dandelion	*In vivo *	0.5 and 2 g/kg dandelion leaf water extract [62]
	Chrysin *Honey, honeycomb* [63]	*In vivo *	20 and 40 mg/kg/d chrysin [63]
	Medium-chain triglycerides (MCTs) *Coconut and coconut oil *	*In vivo *	32% calories as MCTs [64]

**(a):** 

Enzyme	Food, beverage, or bioactive compounds *Food sources in italics *	Type of study	Dosages used and references
CYP3A	Rooibos tea	*In vivo *	Rooibos tea, 4 g/L simmered for 5 minutes, as sole beverage [69]

CYP3A1	Garlic	*In vivo *	30 to 200 mg/kg garlic oil [36] 80 and 200 mg/kg garlic oil 3 times weekly [70]
	Fish oil	*In vivo *	20.5 g/kg fish oil [36]: *note high dose used *

CYP3A2	Garlic	*In vivo *	200 mg/kg diallyl sulfide [8]
	Cruciferous vegetables	*In vivo *	50 mg/kg/d indole-3-carbinol [75]

CYP3A4	Curcumin *Turmeric, curry powder* [34]	*In vivo *	50 and 100 mg/kg curcumin [11]

**(b):** 

Enzyme	Food, beverage, or bioactive compounds *Food sources in italics *	Type of study	Dosages used and references
CYP3A	Green tea	*In vivo *	45 mL/d/rat (avg. 150 g animal weight) green tea [33] 400 mg/kg green tea extract [71] 100 mg/kg/d green tea extract [56]
	Black tea	*In vivo *	54 mL/d/rat (avg. 150 g animal weight) black tea [33]
	Quercetin *Apple, apricot, blueberries, yellow onion, kale, alfalfa sprouts, green beans, broccoli, black tea,* and* chili powder* [47, 48]	*In vivo *	10 and 20 mg/kg [72]

CYP3A2	Cruciferous vegetables	*In vivo *	12 mg/kg/d sulforaphane [57]

	Grapefruit	Clinical	200 mL grapefruit juice 3 times daily [74]
	Resveratrol *Grapes, wine, peanuts, soy, *and *itadori tea* [32]	Clinical	1 g/d resveratrol [28]: *note high dose used *
CYP3A4	Garden cress	Clinical	7.5 g twice daily dose of garden cress seed powder [55]
	Soybean	*In vivo *	100 mg/kg soybean extract [7]
	Kale	*In vivo *	2 g/kg/d kale, as freeze-dried kale drink [51]
	Myricetin *Onions, berries, grapes,* and* red wine* [58]	*In vivo *	0.4, 2, and 8 mg/kg myricetin [58]

**Table 4:** Human and *in vivo* example nutrient inducers of selected CYP4 enzymes.

Enzyme	Food, beverage, or bioactive compounds *Food sources in italics *	Type of study	Dosages used and references
CYP4A1	Green tea	*In vivo *	Green tea (2.5% w/v) as sole beverage [40]

CYP4B1	Caffeic acid *Coffee* [80]	*In vivo *	179 mg/kg caffeic acid [79]

**(a):** 

Enzyme	Food, beverage, or bioactive compounds *Food sources in italics *	Type of study	Dosages used and references
	Cruciferous vegetables	Clinical	Approximately 5 and 10 servings/d of cruciferous vegetables including frozen broccoli, cauliflower, fresh cabbage (red and green), and fresh radish sprouts [90] 250 g/d each of Brussel sprouts and broccoli [25] 2 oz (56.8 g) watercress three times daily [91]
	Resveratrol *Grapes, wine, peanuts, soy, *and *itadori tea* [32]	Clinical	1 g/d resveratrol [28]: *note high dose used *
	Citrus	Observational	0.5+ servings/day of citrus fruits or foods [92]
	Dandelion	*In vivo *	Free access to 2% dandelion tea solution [53]
	Rooibos tea	*In vivo *	Rooibos tea as sole beverage; concentration 2 g tea leaves/100 mL water steeped for 30 minutes [93]
UGTs	Honeybush tea	*In vivo *	Honeybush tea as sole beverage; concentration 4 g tea leaves/100 mL water steeped for 30 minutes [93]
	Rosemary	*In vivo *	Diet of 0.5% rosemary extract [37]
	Soy	*In vivo *	150 and 500 mg/kg soy extract [94]
	Ellagic acid *Berries, pomegranate, grapes, walnuts, *and *blackcurrants* [42]	*In vivo *	Diet of 1% ellagic acid [95]
	Ferulic acid *Whole grains, roasted coffee, tomatoes, asparagus, olives, berries, peas, vegetables,* and* citrus* [96]	*In vivo *	Diet of 1% ferulic acid [95]
	Curcumin *Turmeric, curry powder* [34]	*In vivo *	Diet of 1% curcumin [95]
	Astaxanthin *Algae, yeast, salmon, trout, krill, shrimp,* and* crayfish* [38]	*In vivo *	Diets of 0.001–0.03% astaxanthin for 15 days [39]

**(b):** 

*Legumes *	Mung bean seeds, adzuki bean sprouts [97]

*Vegetables and fruits *	Oranges, spinach, apples, carrots, alfalfa sprouts, cabbage, Brussel sprouts, cauliflower, broccoli, grapefruit, grapes, peaches, plums, lemons, apricots, sweet cherries, corn, cucumber, lettuce, celery, green pepper, tomato, and potatoes [97–99]

**(a):** 

Enzyme	Food, beverage, or bioactive compounds *Food sources in italics *	Type of study	Dosages used and references
SULTs	Caffeine *Coffee, cocoa, black tea,* and *green tea* [111]	*In vivo *	2, 10, and 50 mg/kg caffeine [107]
	Retinoic acid (bioactive form of vitamin A) *Meat (especially liver), fish, egg,* and* dairy products *contain retinol*; apple, apricot, artichokes, arugula, asparagus,* and other plant foods contain provitamin A carotenes [111]	*In vivo *	2, 10, and 50 mg/kg/d retinoic acid suspension in corn oil [108]

**(b):** 

*Animal products *	Fish, shellfish, lamb, beef, chicken, pork, duck, goose, turkey, egg, and cheese

*Legumes *	Lentils, peas, and butter beans

*Grains *	Barley, oatmeal

*Vegetables and fruits *	Cabbage, horseradish, Brussel sprouts, leeks, cress, haricot beans, apricots, peaches, spinach, and watercress

*Nuts and seeds *	Brazil nuts, almonds, peanuts, and walnuts

*Herbs and spices *	Mustard, ginger

**(a):** 

Enzyme	Food, beverage, or bioactive compounds *Food sources in italics *	Type of study	Dosages used and references
	Cruciferous vegetables	Clinical, observational	Approximately 5 and 10 servings/d of cruciferous vegetables including frozen broccoli, cauliflower, fresh cabbage (red and green), and fresh radish sprouts [114] >31.2 g/d cruciferous vegetables [115] 4.5 cups of cruciferous vegetables/d, including 0.5 cups of radish sprouts, 1 cup of frozen cauliflower, 2 cups of frozen broccoli, and 1 cup of fresh cabbage [116] 300 g/d cooked Brussels sprouts [117]
	Allium vegetables	Clinical	3 tbsp fresh chives, 1.33 cups of fresh leeks, 1 tsp garlic, and 0.5 cups of fresh onion [116]
	Resveratrol *Grapes, wine, peanuts, soy, *and *itadori tea* [32]	Clinical	1 g/d resveratrol [28]: *note high dose used *
	Citrus	Observational*, in vivo *	>76 g/d citrus [115] 20 mg limonoid mixture every 2 days [124]
	Garlic	*In vivo *	30 to 200 mg/kg garlic oil [36] 80 and 200 mg/kg garlic oil 3 times weekly [70]
GSTs	Fish oil	*In vivo *	20.5 g/kg fish oil [36]: *note high dose used *
	Black soybean	*In vivo *	1 g/kg black soybean seed coat extract [44]
	Purple sweet potato	*In vivo *	100 and 200 mg/kg anthocyanin extract from purple sweet potato [118]
	Curcumin	*In vivo *	Diet of 2% curcumin [119]
	Green tea	*In vivo *	Equivalent of 4 cups/d (200 mL each) of green tea [120]
	Rooibos tea	*In vivo *	Rooibos tea as sole beverage; concentration 2 g tea leaves/100 mL water steeped for 30 minutes [93]
	Honeybush tea	*In vivo *	Honeybush tea as sole beverage; concentration 4 g tea leaves/100 mL water steeped for 30 minutes [93]
	Ellagic acid *Berries, pomegranate, grapes, walnuts, *and *blackcurrants* [42]	*In vivo *	30 mg/kg/d ellagic acid [43]
	Rosemary	*In vivo *	20 mg/kg carnosic acid 3 times weekly [121]
	Ghee (clarified butter)	*In vivo *	19.5 mg CLA (conjugated linoleic acid)/g fat [122]
	Genistein (kidney GSTs) *Fermented soy *(e.g.,* miso, tempeh*) contains up to 40% bioavailable genistein versus 1% or less in other soy products [125]	*In vivo *	1.5 g/kg genistein [123]: *note high dose used *

**(b):** 

Enzyme	Food, beverage, or bioactive compounds *Food sources in italics *	Type of study	Dosages used and references
	Apiaceous vegetables	Clinical	1 tsp fresh dill weed, 0.5 cups of fresh celery, 3 tbsp. fresh parsley, 1.25 cups of grated parsnips, and 0.75 cups of frozen carrots [116]
GSTs	Quercetin *Apple, apricot, blueberries, yellow onion, kale*, and* alfalfa sprouts, green beans, broccoli, black tea*, and *chili powder* [47, 48]	*In vivo *	2 g/kg quercetin [126]: *note high dose used *
	Genistein (liver GSTs) *Fermented soy *(e.g.,* miso, tempeh*) containsup to 40% bioavailable genistein, versus 1% or less in other soy products [125]	*In vivo *	1.5 g/kg genistein [123]: *note high dose used *

**(c):** 

*Vitamin B6 *	Turkey, pork, chicken, beef, amaranth, lentils, pistachio nuts, sunflower seeds, garlic, and prunes

*Magnesium *	Nuts, seeds, beans, and whole grains

*Selenium *	Brazil nuts, pork, turkey, lamb, chicken, and egg

*Methionine *	Turkey, pork, chicken, beef, egg, Brazil nuts, soybean, sesame seeds, and spirulina

*Cystine *	Pork, turkey, chicken, egg, soybean, spirulina, sesame seeds, and oats

*Glycine *	Turkey, pork, chicken, amaranth, soybean, peanuts, pumpkin seed, and beef

*Folate *(dietary form of folic acid)	Mung bean, adzuki bean, and other legumes, liver, sunflower seeds, quinoa, spinach, asparagus, avocados, mustard greens, and artichokes

*Alpha-lipoic acid *	Spinach, broccoli, tomato, peas, Brussels sprouts, and visceral meats [127, 128]

*Functional foods *	Turmeric, milk thistle, cruciferous vegetables, and artichoke [129–133]

**Table 8:** Amino acids used in phase II conjugation and selected food sources.

*Glycine *	Turkey, pork, chicken, soybean, seaweed, eggs, amaranth, beef, mollusks, peanuts, pumpkin seeds, almonds, duck, goose, mung beans, sunflower seeds, lentils, lamb, bison, lobster, and fish [111]

*Taurine *	Many cooked meats and fish supply taurine. Taurine is also synthesized in the body from cystine (requiring niacin and vitamin B6) and homocysteine (requiring additionally betaine and serine) [144]

*Glutamine *	Plant and animal proteins such as beef, pork, chicken, dairy products, spinach, parsley, and cabbage [145]

*Ornithine *	Ornithine is synthesized endogenously via the urea cycle, requiring arginine and magnesium [144]

*Arginine *	Turkey and pork are especially rich sources; also chicken, pumpkin seeds, soybean, butternuts, egg, peanuts, walnuts, split peas, mollusks, almonds, sesame seeds, lentils, fava beans, mung beans, pine nuts, beef, sunflower seeds, and white beans [111]

**Table 9:** Selected dietary sources of nutrients for methylation support (adapted from [111]).

*Methionine *	Meats, poultry, fish, shellfish, egg, nuts (especially Brazil nuts), seeds (especially sesame seeds and pumpkin seeds), spirulina, teff, soybeans Lower amounts found in other legumes and whole grains (especially teff and oats)

*Vitamin B12 *	Meats and meat products (especially liver and kidney), poultry, fish, shellfish, and eggs

*Vitamin B6 *	Meats, nuts (especially pistachio), garlic, whole grains, seeds (especially sesame and sunflower seeds), legumes (especially chickpeas and lentils), and prunes

*Betaine *	Quinoa, beets, spinach, whole grains (especially rye, kamut, bulgur, amaranth, barley, and oats) sweet potato, meats, and poultry

*Folate *	Beans and legumes (especially mung beans, adzuki beans, chickpeas, and lentils), liver, nuts (especially peanuts), seeds (especially sunflower seeds), spinach, asparagus, mustard greens, and avocado

*Magnesium *	Seeds (especially pumpkin seeds and sesame seeds), beans (especially soybeans), nuts (especially Brazil nuts and almonds), and whole grains (especially amaranth)

**(a):** 

Enzyme	Food, beverage, or bioactive compounds *Food sources in italics *	Type of study	Dosages used and references
	Fish oil	Clinical	3 × 1 g/d fish oil containing 1098 mg EPA and 549 mg DHA [181]
	Lycopene *Tomatoes, rose hips, guava, watermelon,* and *papaya* [111]	Clinical	2 × 15 mg/d lycopene [181]
	Curcumin *Turmeric, curry powder* [34]	*In vivo *	200 mg/kg/d curcumin [155] 75 mg/kg/d curcumin [156] 50 mg/kg/d curcumin [157] 200 mg/kg/d curcumin [158]
	Cruciferous vegetables	*In vivo *	0.5 mg/kg/d sulforaphane [159] Diet of 15% crushed broccoli seed [160]
	Garlic	*In vivo *	50 and 100 mg/kg/d diallyl disulfide [161] 250 mg/kg/d raw garlic [162] 25 mg/kg S-allyl cysteine [163]
	Catechins *Tea *(especially* green tea*)*, cocoa, legumes, *and *grapes* [182]	*In vivo *	5, 15, and 45 mg/kg epicatechin [164] 15 mg/kg epicatechin [165] 20 mg/kg Theaphenon E (95% EGCG) [166] 5, 15, and 30 mg/kg epicatechin [167]
	Resveratrol *Grapes, wine, peanuts, soy, *and *itadori tea* [32]	*In vivo *	10 mg/kg/d [168] 20 mg/kg/d [169]
Nrf2	Ginger	*In vivo *	100 mg/kg/d [6]-shogaol [170] 10 and 100 mg/kg dried ginger extract [171]
	Purple sweet potato	*In vivo *	100 and 200 mg/kg anthocyanin extract from purple sweet potato [118]
	Isoflavones *Soy, kudzu root,* and* red clover* [183]	*In vivo *	80 mg/kg/d soy isoflavones [172] 60 and 120 mg/kg puerarin from kudzu root [173]
	Coffee	*In vivo *	2.0 mL/d coffee to an average animal weight of 200 g ± 10 g [174]
	Rosemary	*In vivo *	50 and 100 mg/kg carnosic acid [175] 5 mg/animal carnosol extract [176]
	Blueberry	*In vivo *	200 mg/kg blueberry [166] 0.6 and 10 g/day [177]
	Pomegranate	*In vivo *	1 and 10 g/kg pomegranate extract [178]: *note high doses used *
	Naringenin *Citrus* [179]	*In vivo *	50 mg/kg/d naringenin [179]
	Ellagic acid *Berries, pomegranate, grapes, walnuts, *and *blackcurrants* [42]	*In vivo *	Diet of 0.4% ellagic acid [166]
	Asthaxanthin *Algae, yeast, salmon, trout, krill, shrimp,* and* crayfish* [38]	*In vivo *	15 mg/kg astaxanthin [166]
	*γ*-tocopherol *Nuts, seeds, whole grains, vegetable oils, *and *legumes* [111]	*In vivo *	20.8 mg/kg *γ*-tocopherol [180]

**(b):** 

Enzyme	Food, beverage, or bioactive compounds	Type of study	Dosages used and references
Nrf2	Luteolin	*In vivo *	40 mg/kg luteolin three times per week [184]
	Quercetin	*In vivo *	50 mg/kg/d quercetin [185]

**Table 11:** Food, beverages, and bioactive compounds with demonstrated, or potential, clinical impact on detoxification systems.

Food or beverage	Nutrient bioactive compounds
Allium vegetables	Astaxanthin
Apiaceous vegetables	Caffeic acid
Black raspberry	Catechins (*including EGCG*)
Black tea	Chrysin
Blueberry	Curcumin
Chamomile tea	Daidzein
Chicory root	Ellagic acid
Citrus	Ferulic acid
Coffee	Fish oil
Cruciferous vegetables (*with potential *	Genistein
*for distinct effects of different *	Luteolin
*crucifers*)	Lycopene
Dandelion tea	MCTs
Garlic	Myricetin
Ghee	N-acetyl cysteine
Ginger	Naringenin
Grapefruit	Quercetin
Green tea	Resveratrol
Honeybush tea	Retinoic acid (*vitamin A*)
Peppermint tea	
Pomegranate	
Purple sweet potato	
Rooibos tea	
Rosemary	
Soybean/black soybean	
Turmeric	

## References

[1] Baer-Dubowska W., Szaefer H. (2013). Modulation of carcinogen-metabolizing cytochromes P450 by phytochemicals in humans. *Expert Opinion on Drug Metabolism and Toxicology*.

[2] Steinkellner H., Rabot S., Freywald C. (2001). Effects of cruciferous vegetables and their constituents on drug metabolizing enzymes involved in the bioactivation of DNA-reactive dietary carcinogens. *Mutation Research*.

[3] Moon Y. J., Wang X., Morris M. E. (2006). Dietary flavonoids: effects on xenobiotic and carcinogen metabolism. *Toxicology in Vitro*.

[4] Hakooz N., Hamdan I. (2007). Effects of dietary broccoli on human in vivo caffeine metabolism: a pilot study on a group of Jordanian volunteers. *Current Drug Metabolism*.

[5] James D., Devaraj S., Bellur P., Lakkanna S., Vicini J., Boddupalli S. (2012). Novel concepts of broccoli sulforaphanes and disease: induction of phase II antioxidant and detoxification enzymes by enhanced-glucoraphanin broccoli. *Nutrition Reviews*.

[6] Aiyer H. S., Gupta R. C. (2010). Berries and ellagic acid prevent estrogen-induced mammary tumorigenesis by modulating enzymes of estrogen metabolism. *Cancer Prevention Research*.

[7] Bogacz A, Mikołajczak P. Ł., Mikołajczak P. Ł. (2014). The influence of soybean extract on the expression level of selected drug transporters, transcription factors and cytochrome P450 genes encoding phase I drug-metabolizing enzymes. *Ginekologia Polska*.

[8] Davenport D. M., Wargovich M. J. (2005). Modulation of cytochrome P450 enzymes by organosulfur compounds from garlic. *Food and Chemical Toxicology*.

[9] Lii C. K., Tsai C. W., Wu C. C. (2006). Garlic allyl sulfides display differential modulation of rat cytochrome P450 2B1 and the placental form glutathione S-transferase in various organs. *Journal of Agricultural and Food Chemistry*.

[10] Kaefer C. M., Milner J. A. (2008). The role of herbs and spices in cancer prevention. *Journal of Nutritional Biochemistry*.

[11] Hsieh Y. W., Huang C. Y., Yang S. Y. (2014). Oral intake of curcumin markedly activated CYP 3A4: in vivo and ex-vivo studies. *Scientific Reports*.

[12] Murray M., Pizzorno J. (1998). *Encyclopedia of Natural Medicine*.

[13] Institute for Functional Medicine (2006). *Textbook of Functional Medicine*.

[14] Ullrich V. (1979). Cytochrome P450 and biological hydroxylation reactions. *Topics in Current Chemistry*.

[15] Danielson P. B. (2002). The cytochrome P450 superfamily: biochemistry, evolution and drug metabolism in humans. *Current Drug Metabolism*.

[16] Paine A. J. (1981). Hepatic cytochrome P-450. *Essays in Biochemistry*.

[17] Chen Q., Zhang T., Wang J. F., Wei D. Q. (2011). Advances in human cytochrome P450 and personalized medicine. *Current Drug Metabolism*.

[18] Ma Q., Lu A. Y. H. (2007). CYP1A induction and human risk assessment: an evolving tale of in vitro and in vivo studies. *Drug Metabolism and Disposition*.

[19] James M. O., Sacco J. C., Faux L. R. (2008). Effects of food natural products on the biotransformation of PCBs. *Environmental Toxicology and Pharmacology*.

[20] Vistisen K., Loft S., Olsen J. H. (2004). Low CYP1A2 activity associated with testicular cancer. *Carcinogenesis*.

[21] Božina N., Bradamante V., Lovrić M. (2009). Genetic polymorphism of metabolic enzymes P450 (CYP) as a susceptibility factor for drug response, toxicity, and cancer risk. *Arhiv za Higijenu Rada i Toksikologiju*.

[22] Tsuchiya Y., Nakajima M., Yokoi T. (2005). Cytochrome P450-mediated metabolism of estrogens and its regulation in human. *Cancer Letters*.

[23] Michnovicz J. J., Bradlow H. L. (1990). Induction of estradiol metabolism by dietary indole-3-carbinol in humans. *Journal of the National Cancer Institute*.

[24] Peterson S., Schwarz Y., Li S. S. (2009). *CYP1A2*, *GSTM1*, and *GSTT1* polymorphisms and diet effects on CYP1A2 activity in a crossover feeding trial. *Cancer Epidemiology Biomarkers and Prevention*.

[25] Walters D. G., Young P. J., Agus C. (2004). Cruciferous vegetable consumption alters the metabolism of the dietary carcinogen 2-amino-1-methyl-6-phenylimidazo[4,5-b]pyridine (PhIP) in humans. *Carcinogenesis*.

[26] Kall M. A., Vang O., Clausen J. (1996). Effects of dietary broccoli on human in vivo drug metabolizing enzymes: evaluation of caffeine, oestrone and chlorzoxazone metabolism. *Carcinogenesis*.

[27] Horn T. L., Reichert M. A., Bliss R. L., Malejka-Giganti D. (2002). Modulations of P450 mRNA in liver and mammary gland and P450 activities and metabolism of estrogen in liver by treatment of rats with indole-3-carbinol. *Biochemical Pharmacology*.

[28] Chow H. H. S., Garland L. L., Hsu C. H. (2010). Resveratrol modulates drug- and carcinogen-metabolizing enzymes in a healthy volunteer study. *Cancer Prevention Research*.

[29] Chen Y., Xiao P., Ou-Yang D. S. (2009). Simultaneous action of the flavonoid quercetin on cytochrome p450 (cyp) 1a2, cyp2a6, n-acetyltransferase and xanthine oxidase activity in healthy volunteers. *Clinical and Experimental Pharmacology and Physiology*.

[30] Lord R. S., Bongiovanni B., Bralley J. A. (2002). Estrogen metabolism and the diet-cancer connection: rationale for assessing the ratio of urinary hydroxylated estrogen metabolites. *Alternative Medicine Review*.

[31] Takemura H., Sakakibara H., Yamazaki S., Shimoi K. (2013). Breast cancer and flavonoids—a role in prevention. *Current Pharmaceutical Design*.

[32] Burns J., Yokota T., Ashihara H., Lean M. E. J., Crozier A. (2002). Plant foods and herbal sources of resveratrol. *Journal of Agricultural and Food Chemistry*.

[33] Yao H. T., Hsu Y. R., Lii C. K., Lin A. H., Chang K. H., Yang H. T. (2014). Effect of commercially available green and black tea beverages on drug-metabolizing enzymes and oxidative stress in Wistar rats. *Food and Chemical Toxicology*.

[34] Tayyem R. F., Heath D. D., Al-Delaimy W. K., Rock C. L. (2006). Curcumin content of turmeric and curry powders. *Nutrition and Cancer*.

[35] Bansal S. S., Kausar H., Vadhanam M. V. (2014). Curcumin implants, not curcumin diet, inhibit estrogen-induced mammary carcinogenesis in ACI rats. *Cancer Prevention Research*.

[36] Chen H. W., Tsai C. W., Yang J. J., Liu C. T., Kuo W. W., Lii C. K. (2003). The combined effects of garlic oil and fish oil on the hepatic antioxidant and drug-metabolizing enzymes of rats. *British Journal of Nutrition*.

[37] Debersac P., Heydel J. M., Amiot M. J. (2001). Induction of cytochrome P450 and/or detoxication enzymes by various extracts of rosemary: Description of specific patterns. *Food and Chemical Toxicology*.

[38] Ambati R. R., Moi P. S., Ravi S., Aswathanarayana R. G. (2014). Astaxanthin: sources, extraction, stability, biological activities and its commercial applications—a review. *Marine Drugs*.

[39] Gradelet S., Astorg P., Leclerc J., Chevalier J., Vernevaut M.-F., Siess M.-H. (1996). Effects of canthaxanthin, astaxanthin, lycopene and lutein on liver xenobiotic-metabolizing enzymes in the rat. *Xenobiotica*.

[40] Bu-Abbas A., Clifford M. N., Walker R., Ioannides C. (1994). Selective induction of rat hepatic CYP1 and CYP4 proteins and of peroxisomal proliferation by green tea. *Carcinogenesis*.

[41] Rasmussen M. K., Brunius C., Zamaratskaia G., Ekstrand B. (2012). Feeding dried chicory root to pigs decrease androstenone accumulation in fat by increasing hepatic 3*β* hydroxysteroid dehydrogenase expression. *Journal of Steroid Biochemistry and Molecular Biology*.

[42] Usta C., Ozdemir S., Schiariti M., Puddu P. E. (2013). The pharmacological use of ellagic acid-rich pomegranate fruit. *International Journal of Food Sciences and Nutrition*.

[43] Celik G., Semiz A., Karakurt S., Arslan S., Adali O., Sen A. (2013). A comparative study for the evaluation of two doses of ellagic acid on hepatic drug metabolizing and antioxidant enzymes in the rat. *BioMed Research International*.

[44] Zhang T., Jiang S., He C., Kimura Y., Yamashita Y., Ashida H. (2013). Black soybean seed coat polyphenols prevent B(a)P-induced DNA damage through modulating drug-metabolizing enzymes in HepG2 cells and ICR mice. *Mutation Research*.

[45] Catterall F., McArdle N. J., Mitchell L., Papayanni A., Clifford M. N., Ioannides C. (2003). Hepatic and intestinal cytochrome P450 and conjugase activities in rats treated with black tea theafulvins and theaflavins. *Food and Chemical Toxicology*.

[46] Thapliyal R., Maru G. B. (2001). Inhibition of cytochrome P450 isozymes by curcumins in vitro and in vivo. *Food and Chemical Toxicology*.

[47] Sampson L., Rimm E., Hollman P. C. H., de Vries J. H. M., Katan M. B. (2002). Flavonol and flavone intakes in US health professionals. *Journal of the American Dietetic Association*.

[48] Hertog M. G. L., Feskens E. J. M., Hollman P. C. H. (1992). Content of potentially anticarcinogenic flavonoids of 28 vegetables and 9 fruits commonly consumed in the Netherlands. *Journal of Agricultural and Food Chemistry*.

[49] Peng W. X., Li H. D., Zhou H. H. (2003). Effect of daidzein on CYP1A2 activity and pharmacokinetics of theophylline in healthy volunteers. *European Journal of Clinical Pharmacology*.

[50] Fuhr U., Klittich K., Staib A. H. (1993). Inhibitory effect of grapefruit juice and its bitter principal, naringenin, on CYP1A2 dependent metabolism of caffeine in man. *British Journal of Clinical Pharmacology*.

[51] Yamasaki I., Yamada M., Uotsu N., Teramoto S., Takayanagi R., Yamada Y. (2012). Inhibitory effects of kale ingestion on metabolism by cytochrome P450 enzymes in rats. *Biomedical Research*.

[52] Zeng T., Zhang C. L., Song F. Y., Han X. Y., Xie K. Q. (2009). The modulatory effects of garlic oil on hepatic cytochrome P450s in mice. *Human and Experimental Toxicology*.

[53] Maliakal P. P., Wanwimolruk S. (2001). Effect of herbal teas on hepatic drug metabolizing enzymes in rats. *Journal of Pharmacy and Pharmacology*.

[54] Nissar A. U., Farrukh M. R., Kaiser P. J. (2013). Effect of N-acetyl cysteine (NAC), an organosulfur compound from Allium plants, on experimentally induced hepatic prefibrogenic events in wistar rat. *Phytomedicine*.

[55] Al-Jenoobi F. I., Al-Thukair A. A., Alam M. A. (2014). Effect of garden cress seeds powder and its alcoholic extract on the metabolic activity of CYP2D6 and CYP3A4. *Evidence-Based Complementary and Alternative Medicine*.

[56] Park D., Jeon J. H., Shin S. (2009). Green tea extract increases cyclophosphamide-induced teratogenesis by modulating the expression of cytochrome P-450 mRNA. *Reproductive Toxicology*.

[57] Yoxall V., Kentish P., Coldham N., Kuhnert N., Sauer M. J., Ioannides C. (2005). Modulation of hepatic cytochromes P450 and phase II enzymes by dietary doses of sulforaphane in rats: implications for its chemopreventive activity. *International Journal of Cancer*.

[58] Li C., Lim S. C., Kim J., Choi J. S. (2011). Effects of myricetin, an anticancer compound, on the bioavailability and pharmacokinetics of tamoxifen and its main metabolite, 4-hydroxytamoxifen, in rats. *European Journal of Drug Metabolism and Pharmacokinetics*.

[59] Leclercq I., Desager J. P., Horsmans Y. (1998). Inhibition of chlorzoxazone metabolism, a clinical probe for CYP2E1, by a single ingestion of watercress. *Clinical Pharmacology and Therapeutics*.

[60] Loizou G. D., Cocker J. (2001). The effects of alcohol and diallyl sulphide on CYP2E1 activity in humans: a phenotyping study using chlorzoxazone. *Human and Experimental Toxicology*.

[61] Park K. A., Kweon S., Choi H. (2002). Anticarcinogenic effect and modification of cytochrome P450 2E1 by dietary garlic powder in diethylnitrosamine-initiated rat hepatocarcinogenesis. *Journal of Biochemistry and Molecular Biology*.

[62] Park C. M., Cha Y. S., Youn H. J., Cho C. W., Song Y. S. (2010). Amelioration of oxidative stress by dandelion extract through CYP2E1 suppression against acute liver injury induced by carbon tetrachloride in sprague-dawley rats. *Phytotherapy Research*.

[63] Tahir M., Sultana S. (2011). Chrysin modulates ethanol metabolism in Wistar rats: a promising role against organ toxicities. *Alcohol and Alcoholism*.

[64] Lieber C. S., Cao Q., Decarli L. M. (2007). Role of medium-chain triglycerides in the alcohol-mediated cytochrome P450 2E1 induction of mitochondria. *Alcoholism: Clinical and Experimental Research*.

[65] Sheweita S. A. (2000). Drug-metabolizing enzymes: mechanisms and functions. *Current Drug Metabolism*.

[66] Zgheib N. K., Mitri Z., Geryess E., Noutsi P. (2010). Cytochrome P4502E1 (CYP2E1) genetic polymorphisms in a Lebanese population: frequency distribution and association with morbid diseases. *Genetic Testing and Molecular Biomarkers*.

[67] González C. A., Sala N., Capellá G. (2002). Genetic susceptibility and gastric cancer risk. *International Journal of Cancer*.

[68] Armoni M., Harel C., Ramdas M., Karnieli E. (2014). CYP2E1 impairs GLUT4 gene expression and function: NRF2 as a possible mediator. *Hormone and Metabolic Research*.

[69] Matsuda K., Nishimura Y., Kurata N., Iwase M., Yasuhara H. (2007). Effects of continuous ingestion of herbal teas on intestinal CYP3A in the rat. *Journal of Pharmacological Sciences*.

[70] Wu C. C., Sheen L. Y., Chen H. W., Kuo W. W., Tsai S. J., Lii C. K. (2002). Differential effects of garlic oil and its three major organosulfur components on the hepatic detoxification system in rats. *Journal of Agricultural and Food Chemistry*.

[71] Misaka S., Kawabe K., Onoue S. (2013). Green tea extract affects the cytochrome P450 3A activity and pharmacokinetics of simvastatin in rats. *Drug Metabolism and Pharmacokinetics*.

[72] Umathe S. N., Dixit P. V., Kumar V., Bansod K. U., Wanjari M. M. (2008). Quercetin pretreatment increases the bioavailability of pioglitazone in rats: involvement of CYP3A inhibition. *Biochemical Pharmacology*.

[73] Liu J., Tawa G. J., Wallqvist A. (2013). Identifying cytochrome P450 functional networks and their allosteric regulatory elements. *PLoS ONE*.

[74] Tanaka S., Uchida S., Miyakawa S. (2013). Comparison of inhibitory duration of grapefruit juice on organic anion-transporting polypeptide and cytochrome P450 3A4. *Biological and Pharmaceutical Bulletin*.

[75] Leibelt D. A., Hedstrom O. R., Fisher K. A., Pereira C. B., Williams D. E. (2003). Evaluation of chronic dietary exposure to indole-3-carbinol and absorption-enhanced 3,3′-diidolylmethane in Sprague-Dawley rats. *Toxicological Sciences*.

[76] Linus Pauling Institute (2008). *Garlic and Organosulfur Compounds*.

[77] Ioannides C. (1999). Effect of diet and nutrition on the expression of cytochromes P450. *Xenobiotica*.

[78] Baer B., Rettie A. (2006). CYP4B1: an enigmatic P450 at the interface between xenobiotic and endobiotic metabolism. *Drug Metabolism Reviews*.

[79] Ye Z., Liu Z., Henderson A. (2009). Increased CYP4B1 mRNA is associated with the inhibition of dextran sulfate sodium-induced colitis by caffeic acid in mice. *Experimental Biology and Medicine (Maywood)*.

[80] Lafay S., Morand C., Manach C., Besson C., Scalbert A. (2006). Absorption and metabolism of caffeic acid and chlorogenic acid in the small intestine of rats. *British Journal of Nutrition*.

[81] Xu C., Li C. Y., Kong A. T. (2005). Induction of phase I, II and III drug metabolism/transport by xenobiotics. *Archives of Pharmacal Research*.

[82] Ginsberg G., Guyton K., Johns D., Schimek J., Angle K., Sonawane B. (2010). Genetic polymorphism in metabolism and host defense enzymes: implications for human health risk assessment. *Critical Reviews in Toxicology*.

[83] Jancova P., Anzenbacher P., Anzenbacherova E. (2010). Phase II drug metabolizing enzymes. *Biomedical Papers*.

[84] Jenkinson C., Petroczi A., Naughton D. P. (2013). Effects of dietary components on testosterone metabolism via UDP-glucuronosyltransferase. *Frontiers in Endocrinology*.

[85] Chang J. L., Bigler J., Schwarz Y. (2007). UGT1A1 polymorphism is associated with serum bilirubin concentrations in a randomized, controlled, fruit and vegetable feeding trial. *Journal of Nutrition*.

[86] Rowland A., Miners J. O., Mackenzie P. I. (2013). The UDP-glucuronosyltransferases: their role in drug metabolism and detoxification. *International Journal of Biochemistry and Cell Biology*.

[87] Strassburg C. P., Kneip S., Topp J. (2000). Polymorphic gene regulation and interindividual variation of UDP-glucuronosyltransferase activity in human small intestine. *The Journal of Biological Chemistry*.

[88] Wells P. G., Mackenzie P. I., Chowdhury J. R. (2004). Glucuronidation and the UDP-glucuronosyltransferases in health and disease. *Drug Metabolism and Disposition*.

[89] Lampe J. W. (2009). Interindividual differences in response to plant-based diets: Implications for cancer risk. *The American Journal of Clinical Nutrition*.

[90] Navarro S. L., Peterson S., Chen C. (2009). Cruciferous vegetable feeding alters UGT1A1 activity: diet- and genotype-dependent changes in serum bilirubin in a controlled feeding trial. *Cancer Prevention Research (Phila)*.

[91] Hecht S. S., Carmella S. G., Murphy S. E. (1999). Effects of watercress consumption on urinary metabolites of nicotine in smokers. *Cancer Epidemiology Biomarkers and Prevention*.

[92] Saracino M. R., Bigler J., Schwarz Y. (2009). Citrus fruit intake is associated with lower serum bilirubin concentration among women with the UGT1A1^*^28 polymorphism. *Journal of Nutrition*.

[93] Marnewick J. L., Joubert E., Swart P., van der Westhuizen F., Gelderblom W. C. (2003). Modulation of hepatic drug metabolizing enzymes and oxidative status by rooibos (*Aspalathus linearis*) and Honeybush (*Cyclopia intermedia*), green and black (*Camellia sinensis*) teas in rats. *Journal of Agricultural and Food Chemistry*.

[94] Marahatta A., Bhandary B., Jeong S.-K., Kim H.-R., Chae H.-J. (2014). Soybean greatly reduces valproic acid plasma concentrations: a food-drug interaction study. *Scientific Reports*.

[95] van der Logt E. M. J., Roelofs H. M. J., Nagengast F. M., Peters W. H. M. (2003). Induction of rat hepatic and intestinal UDP-glucuronosyltransferases by naturally occurring dietary anticarcinogens. *Carcinogenesis*.

[96] Graf E. (1992). Antioxidant potential of ferulic acid. *Free Radical Biology and Medicine*.

[97] Simone C. B., Simone N. L., Pallante M., Simone C. B. (2001). Cancer, lifestyle modification and glucarate. *Journal of Orthomolecular Medicine*.

[98] Zółtaszek R., Hanausek M., Kiliańska Z. M., Walaszek Z. (2008). The biological role of D-glucaric acid and its derivatives: potential use in medicine. *Postpy Higieny i Medycyny Doświadczalnej*.

[99] Dwivedi C., Heck W. J., Downie A. A., Larroya S., Webb T. E. (1990). Effect of calcium glucarate on beta-glucoronidase activity and glucarate content of certain vegetable and fruits. *Biochemical Medicine and Metabolic Biology*.

[100] Kosmala M., Zduńczyk Z., Kołodziejczyk K., Klimczak E., Jukiewicz J., Zduńczyk P. (2014). Chemical composition of polyphenols extracted from strawberry pomace and their effect on physiological properties of diets supplemented with different types of dietary fibre in rats. *European Journal of Nutrition*.

[101] Maruti S. S., Chang J. L., Prunty J. A. (2008). Serum *β*-glucuronidase activity in response to fruit and vegetable supplementation: a controlled feeding study. *Cancer Epidemiology Biomarkers and Prevention*.

[102] Jurgoński A., Juśkiewicz J., Zduńczyk Z., Matusevicius P., Kołodziejczyk K. (2014). Polyphenol-rich extract from blackcurrant pomace attenuates the intestinal tract and serum lipid changes induced by a high-fat diet in rabbits. *European Journal of Nutrition*.

[103] James M. O., Ambadapadi S. (2013). Interactions of cytosolic sulfotransferases with xenobiotics. *Drug Metabolism Reviews*.

[104] Kodama S., Negishi M. (2013). Sulfotransferase genes: regulation by nuclear receptors in response to xeno/endo-biotics. *Drug Metabolism Reviews*.

[105] Wang L.-Q., James M. O. (2006). Inhibition of sulfotransferases by xenobiotics. *Current Drug Metabolism*.

[106] Ung D., Nagar S. (2007). Variable sulfation of dietary polyphenols by recombinant human sulfotransferase (SULT) 1A1 genetic variants and SULT1E1. *Drug Metabolism and Disposition*.

[107] Zhou T., Chen Y., Huang C., Chen G. (2012). Caffeine induction of sulfotransferases in rat liver and intestine. *Journal of Applied Toxicology*.

[108] Maiti S., Chen X., Chen G. (2005). All-trans retinoic acid induction of sulfotransferases. *Basic and Clinical Pharmacology and Toxicology*.

[109] Kamio K., Honke K., Makita A. (1995). Pyridoxal 5'-phosphate binds to a lysine residue in the adenosine 3′-phosphate 5′-phosphosulfate recognition site of glycolipid sulfotransferase from human renal cancer cells. *Glycoconjugate Journal*.

[110] Masters M., McCance R. A. (1939). The sulfur content of foods. *Biochemical Journal*.

[111] USDA National Nutrient Database for Standard Reference (2011). *Nutrient Data Laboratory. Release 27*.

[112] McFadden S. A. (1996). Phenotypic variation in xenobiotic metabolism and adverse environmental response: focus on sulfur-dependent detoxification pathways. *Toxicology*.

[113] Hayes J. D., Pulford D. J. (1995). The glutathione S-transferase supergene family: regulation of GST and the contribution of the isoenzymes to cancer chemoprotection and drug resistance. *Critical Reviews in Biochemistry and Molecular Biology*.

[114] Navarro S. L., Chang J. L., Peterson S. (2009). Modulation of human serum glutathione *S*-transferase A1/2 concentration by cruciferous vegetables in a controlled feeding study is influenced by *GSTM1* and *GSTT1* genotypes. *Cancer Epidemiology Biomarkers and Prevention*.

[115] Wark P. A., Grubben M. J. A. L., Peters W. H. M. (2004). Habitual consumption of fruits and vegetables: associations with human rectal glutathione S-transferase. *Carcinogenesis*.

[116] Lampe J. W., Chen C., Li S. (2000). Modulation of human glutathione S-transferases by botanically defined vegetable diets. *Cancer Epidemiology Biomarkers and Prevention*.

[117] Nijhoff W. A., Mulder T. P. J., Verhagen H., van Poppel G., Peters W. H. M. (1995). Effects of consumption of brussels sprouts on plasma and urinary glutathione S-transferase class-alpha and -pi in humans. *Carcinogenesis*.

[118] Hwang Y. P., Choi J. H., Yun H. J. (2011). Anthocyanins from purple sweet potato attenuate dimethylnitrosamine-induced liver injury in rats by inducing Nrf2-mediated antioxidant enzymes and reducing COX-2 and iNOS expression. *Food and Chemical Toxicology*.

[119] Iqbal M., Sharma S. D., Okazaki Y., Fujisawa M., Okada S. (2003). Dietary supplementation of curcumin enhances antioxidant and phase II metabolizing enzymes in ddY male mice: possible role in protection against chemical carcinogenesis and toxicity. *Pharmacology and Toxicology*.

[120] Newsome B. J., Petriello M. C., Han S. G. (2014). Green tea diet decreases PCB 126-induced oxidative stress in mice by up-regulating antioxidant enzymes. *Journal of Nutritional Biochemistry*.

[121] Lin C. Y., Chen J. H., Fu R. H., Tsai C. W. (2014). Induction of Pi form of glutathione S-transferase by carnosic acid is mediated through PI3K/Akt/NF-*κ*B pathway and protects against neurotoxicity. *Chemical Research in Toxicology*.

[122] Chinnadurai K., Kanwal H. K., Tyagi A. K., Stanton C., Ross P. (2013). High conjugated linoleic acid enriched ghee (clarified butter) increases the antioxidant and antiatherogenic potency in female Wistar rats. *Lipids in Health and Disease*.

[123] Froyen E. B., Reeves J. L. R., Mitchell A. E., Steinberg F. M. (2009). Regulation of phase II enzymes by Genistein and daidzein in male and female Swiss Webster mice. *Journal of Medicinal Food*.

[124] Perez J. L., Jayaprakasha G. K., Cadena A., Martinez E., Ahmad H., Patil B. S. (2010). In vivo induction of phase II detoxifying enzymes, glutathione transferase and quinone reductase by citrus triterpenoids. *BMC Complementary and Alternative Medicine*.

[125] Barrett J. R. (2006). The science of soy: what do we really know?. *Environmental Health Perspectives*.

[126] Wiegand H., Boesch-Saadatmandi C., Regos I., Treutter D., Wolffram S., Rimbach G. (2009). Effects of quercetin and catechin on hepatic glutathione-s transferase (GST), NAD(P)H quinone oxidoreductase 1 (NQO1), and antioxidant enzyme activity levels in rats. *Nutrition and Cancer*.

[127] Gomes M. B., Negrato C. A. (2014). Alpha-lipoic acid as a pleiotropic compound with potential therapeutic use in diabetes and other chronic diseases. *Diabetology & Metabolic Syndrome*.

[128] Linus Pauling Institute (2012). *Lipoic Acid*.

[129] Kalpravidh R. W., Siritanaratkul N., Insain P. (2010). Improvement in oxidative stress and antioxidant parameters in beta-thalassemia/Hb E patients treated with curcuminoids. *Clinical Biochemistry*.

[130] Lucena M. I., Andrade R. J., de la Cruz J. P., Rodriguez-Mendizabal M., Blanco E., Sánchez de la Cuesta F. (2002). Effects of silymarin MZ-80 on oxidative stress in patients with alcoholic cirrhosis. *International Journal of Clinical Pharmacology and Therapeutics*.

[131] Santana-Martínez R. A., Galván-Arzáte S., Hernández-Pando R. (2014). Sulforaphane reduces the alterations induced by quinolinic acid: modulation of glutathione levels. *Neuroscience*.

[132] Chen M. F., Chen L. T., Boyce H. W. (1995). Cruciferous vegetables and glutathione: their effects on colon mucosal glutathione level and colon tumor development in rats induced by DMH. *Nutrition and Cancer*.

[133] El Morsy E. M., Kamel R. (2015). Protective effect of artichoke leaf extract against paracetamol-induced hepatotoxicity in rats. *Pharmaceutical Biology*.

[134] Brauer H. A., Libby T. E., Mitchell B. L. (2011). Cruciferous vegetable supplementation in a controlled diet study alters the serum peptidome in a GSTM1-genotype dependent manner. *Nutrition Journal*.

[135] Hofmann T., Kuhnert A., Schubert A. (2009). Modulation of detoxification enzymes by watercress: *in vitro* and *in vivo* investigations in human peripheral blood cells. *European Journal of Nutrition*.

[136] Forman H. J., Zhang H., Rinna A. (2009). Glutathione: overview of its protective roles, measurement, and biosynthesis. *Molecular Aspects of Medicine*.

[137] Kern J. K., Geier D. A., Adams J. B., Garver C. R., Audhya T., Geier M. R. (2011). A clinical trial of glutathione supplementation in autism spectrum disorders. *Medical Science Monitor*.

[138] Paterson P. G., Lyon A. W., Kamencic H., Andersen L. B., Juurlink B. H. J. (2001). Sulfur amino acid deficiency depresses brain glutathione concentration. *Nutritional Neuroscience*.

[139] Treweeke A. T., Winterburn T. J., Mackenzie I. (2012). N-Acetylcysteine inhibits platelet-monocyte conjugation in patients with type 2 diabetes with depleted intraplatelet glutathione: a randomised controlled trial. *Diabetologia*.

[140] Galluzzi L., Vitale I., Senovilla L. (2012). Prognostic impact of vitamin B6 metabolism in lung cancer. *Cell Reports*.

[141] Howard J. M., Davies S., Hunnisett A. (1994). Red cell magnesium and glutathione peroxidase in infertile women—effects of oral supplementation with magnesium and selenium. *Magnesium Research*.

[142] Child D. F., Hudson P. R., Jones H. (2004). The effect of oral folic acid on glutathione, glycaemia and lipids in type 2 diabetes. *Diabetes, Nutrition and Metabolism—Clinical and Experimental*.

[143] Ansar H., Mazloom Z., Kazemi F., Hejazi N. (2011). Effect of alpha-lipoic acid on blood glucose, insulin resistance, and glutathione peroxidase of type 2 diabetic patients. *Saudi Medical Journal*.

[144] Lord R. S., Bralley J. A. (2012). *Laboratory Evaluations for Integrative and Functional Medicine*.

[145] University of Maryland Medical Center http://umm.edu/health/medical/altmed/supplement/glutamine.

[146] Makarova S. I. (2008). Human N-acetyltransferases and drug-induced hepatotoxicity. *Current Drug Metabolism*.

[147] Kohalmy K., Vrzal R. (2011). Regulation of phase II biotransformation enzymes by steroid hormones. *Current Drug Metabolism*.

[148] Yager J. D. (2014). Mechanisms of estrogen carcinogenesis: the role of E2/E1-quinone metabolites suggests new approaches to preventive intervention—a review. *Steroids*.

[149] Busserolles J., Zimowska W., Rock E., Rayssiguier Y., Mazur A. (2002). Rats fed a high sucrose diet have altered heart antioxidant enzyme activity and gene expression. *Life Sciences*.

[150] Su Z. Y., Shu L., Khor T. O., Lee J. H., Fuentes F., Kong A. N. T. (2013). A perspective on dietary phytochemicals and cancer chemoprevention: oxidative stress, Nrf2, and epigenomics. *Topics in Current Chemistry*.

[151] Chan K., Han X. D., Kan Y. W. (2001). An important function of Nrf2 in combating oxidative stress: detoxification of acetaminophen. *Proceedings of the National Academy of Sciences of the United States of America*.

[152] Calabrese V., Cornelius C., Mancuso C. (2008). Cellular stress response: A novel target for chemoprevention and nutritional neuroprotection in aging, neurodegenerative disorders and longevity. *Neurochemical Research*.

[153] Boyanapalli S. S., Paredes-Gonzalez X., Fuentes F. (2014). Nrf2 knockout attenuates the anti-inflammatory effects of phenethyl isothiocyanate and curcumin. *Chemical Research in Toxicology*.

[154] Niture S. K., Khatri R., Jaiswal A. K. (2014). Regulation of Nrf2—an update. *Free Radical Biology and Medicine*.

[155] Xie Y., Zhao Q. Y., Li H. Y., Zhou X., Liu Y., Zhang H. (2014). Curcumin ameliorates cognitive deficits heavy ion irradiation-induced learning and memory deficits through enhancing of Nrf2 antioxidant signaling pathways. *Pharmacology Biochemistry and Behavior*.

[156] Soetikno V., Sari F. R., Lakshmanan A. P. (2013). Curcumin alleviates oxidative stress, inflammation, and renal fibrosis in remnant kidney through the Nrf2-keap1 pathway. *Molecular Nutrition & Food Research*.

[157] He H. J., Wang G. Y., Gao Y., Ling W. H., Yu Z. W., Jin T. R. (2012). Curcumin attenuates Nrf2 signaling defect, oxidative stress in muscle and glucose intolerance in high fat diet-fed mice. *World Journal of Diabetes*.

[158] Farombi E. O., Shrotriya S., Na H. K., Kim S. H., Surh Y. J. (2008). Curcumin attenuates dimethylnitrosamine-induced liver injury in rats through Nrf2-mediated induction of heme oxygenase-1. *Food and Chemical Toxicology*.

[159] Zhang Z., Wang S., Zhou S. (2014). Sulforaphane prevents the development of cardiomyopathy in type 2 diabetic mice probably by reversing oxidative stress-induced inhibition of LKB1/AMPK pathway. *Journal of Molecular and Cellular Cardiology*.

[160] McWalter G. K., Higgins L. G., McLellan L. I. (2004). Transcription factor Nrf2 is essential for induction of NAD(P)H:quinone oxidoreductase 1, glutathione S-transferases, and glutamate cysteine ligase by broccoli seeds and isothiocyanates. *Journal of Nutrition*.

[161] Lee I. C., Kim S. H., Baek H. S. (2014). The involvement of Nrf2 in the protective effects of diallyl disulfide on carbon tetrachloride-induced hepatic oxidative damage and inflammatory response in rats. *Food and Chemical Toxicology*.

[162] Padiya R., Chowdhury D., Borkar R., Srinivas R., Pal Bhadra M., Banerjee S. K. (2014). Garlic attenuates cardiac oxidative stress via activation of PI3K/AKT/Nrf2-Keap1 pathway in fructose-fed diabetic rat. *PLoS ONE*.

[163] Gómez-Sierra T., Molina-Jijón E., Tapia E. (2014). S-allylcysteine prevents cisplatin-induced nephrotoxicity and oxidative stress. *Journal of Pharmacy and Pharmacology*.

[164] Chang C. F., Cho S., Wang J. (2014). (-)-Epicatechin protects hemorrhagic brain via synergistic Nrf2 pathways. *Annals of Clinical and Translational Neurology*.

[165] Leonardo C. C., Agrawal M., Singh N., Moore J. R., Biswal S., Doré S. (2013). Oral administration of the flavanol (-)-epicatechin bolsters endogenous protection against focal ischemia through the Nrf2 cytoprotective pathway. *European Journal of Neuroscience*.

[166] Kavitha K., Thiyagarajan P., Rathna J., Mishra R., Nagini S. (2013). Chemopreventive effects of diverse dietary phytochemicals against DMBA-induced hamster buccal pouch carcinogenesis via the induction of Nrf2-mediated cytoprotective antioxidant, detoxification, and DNA repair enzymes. *Biochimie*.

[167] Shah Z. A., Li R.-C., Ahmad A. S. (2010). The flavanol (−)-epicatechin prevents stroke damage through the Nrf2/HO1 pathway. *Journal of Cerebral Blood Flow and Metabolism*.

[168] Tamaki N., Cristina Orihuela-Campos R., Inagaki Y., Fukui M., Nagata T., Ito H. (2014). Resveratrol improves oxidative stress and prevents the progression of periodontitis via the activation of the Sirt1/AMPK and the Nrf2/antioxidant defense pathways in a rat periodontitis model. *Free Radical Biology and Medicine*.

[169] Sadi G., Bozan D., Yildiz H. B. (2014). Redox regulation of antioxidant enzymes: post-translational modulation of catalase and glutathione peroxidase activity by resveratrol in diabetic rat liver. *Molecular and Cellular Biochemistry*.

[170] Chen H., Fu J., Hu Y. (2014). Ginger compound [6]-shogaol and its cysteine-conjugated metabolite (M2) activate Nrf2 in colon epithelial cells *in vitro* and *in vivo*. *Chemical Research in Toxicology*.

[171] Bak M. J., Ok S., Jun M., Jeong W. S. (2012). 6-shogaol-rich extract from ginger up-regulates the antioxidant defense systems in cells and mice. *Molecules*.

[172] Xi Y. D., Li X. Y., Yu H. L. (2014). Soy isoflavone antagonizes the oxidative cerebrovascular injury induced by *β*-Amyloid Peptides 1–42 in Rats. *Neurochemical Research*.

[173] Li R., Liang T., Xu L., Zheng N., Zhang K., Duan X. (2013). Puerarin attenuates neuronal degeneration in the substantia nigra of 6-OHDA-lesioned rats through regulating BDNF expression and activating the Nrf2/ARE signaling pathway. *Brain Research*.

[174] Vicente S. J. V., Ishimoto E. Y., Torres E. A. F. S. (2014). Coffee modulates transcription factor Nrf2 and highly increases the activity of antioxidant enzymes in rats. *Journal of Agricultural and Food Chemistry*.

[175] Sahu B. D., Putcha U. K., Kuncha M., Rachamalla S. S., Sistla R. (2014). Carnosic acid promotes myocardial antioxidant response and prevents isoproterenol-induced myocardial oxidative stress and apoptosis in mice. *Molecular and Cellular Biochemistry*.

[176] Balstad T. R., Carlsen H., Myhrstad M. C. W. (2011). Coffee, broccoli and spices are strong inducers of electrophile response element-dependent transcription in vitro and in vivo—studies in electrophile response element transgenic mice. *Molecular Nutrition and Food Research*.

[177] Wang Y. P., Cheng M. L., Zhang B. F. (2010). Effect of blueberry on hepatic and immunological functions in mice. *Hepatobiliary and Pancreatic Diseases International*.

[178] Bishayee A., Bhatia D., Thoppil R. J., Darvesh A. S., Nevo E., Lansky E. P. (2011). Pomegranate-mediated chemoprevention of experimental hepatocarcinogenesis involves Nrf2-regulated antioxidant mechanisms. *Carcinogenesis*.

[179] Esmaeili M. A., Alilou M. (2014). Naringenin attenuates CCl4-induced hepatic inflammation by the activation of an Nrf2-mediated pathway in rats. *Clinical and Experimental Pharmacology and Physiology*.

[180] Singh C. K., Ndiaye M. A., Siddiqui I. A. (2014). Methaneseleninic acid and *γ*-tocopherol combination inhibits prostate tumor growth in vivo in a xenograft mouse model. *Oncotarget*.

[181] Magbanua M. J. M., Roy R., Sosa E. V. (2011). Gene expression and biological pathways in tissue of men with prostate cancer in a randomized clinical trial of lycopene and fish oil supplementation. *PLoS ONE*.

[182] Manach C., Scalbert A., Morand C., Rémésy C., Jiménez L. (2004). Polyphenols: food sources and bioavailability. *The American Journal of Clinical Nutrition*.

[183] Delmonte P., Rader J. I. (2006). Analysis of isoflavones in foods and dietary supplements. *Journal of AOAC International*.

[184] Chian S., Thapa R., Chi Z., Wang X. J., Tang X. (2014). Luteolin inhibits the Nrf2 signaling pathway and tumor growth in vivo. *Biochemical and Biophysical Research Communications*.

[185] Marina R., González P., Ferreras M. C., Costilla S., Barrio J. P. (2015). Hepatic Nrf2 expression is altered by quercetin supplementation in Xirradiated rats. *Molecular Medicine Reports*.

[186] Zhou H., Qu Z., Mossine V. V. (2014). Proteomic analysis of the effects of aged garlic extract and its fruarg component on lipopolysaccharide-induced neuroinflammatory response in microglial cells. *PLoS ONE*.

[187] Calabrese V., Cornelius C., Dinkova-Kostova A. T., Calabrese E. J., Mattson M. P. (2010). Cellular stress responses, the hormesis paradigm, and vitagenes: novel targets for therapeutic intervention in neurodegenerative disorders. *Antioxidants & Redox Signaling*.

[188] Houghton C. A., Fassett R. G., Coombes J. S. (2013). Sulforaphane: translational research from laboratory bench to clinic. *Nutrition Reviews*.

[189] Stefanson A. L., Bakovic M. (2014). Dietary regulation of Keap1/Nrf2/ARE pathway: focus on plant-derived compounds and trace minerals. *Nutrients*.

[190] Andrews G. K. (2000). Regulation of metallothionein gene expression by oxidative stress and metal ions. *Biochemical Pharmacology*.

[191] Lichtlen P., Schaffner W. (2001). Putting its fingers on stressful situations: the heavy metal-regulatory transcription factor MTF-1. *BioEssays*.

[192] Sato M., Kondoh M. (2002). Recent studies on metallothionein: protection against toxicity of heavy metals and oxygen free radicals. *Tohoku Journal of Experimental Medicine*.

[193] Pan Y., Huang J., Xing R. (2013). Metallothionein 2A inhibits NF-*κ*B pathway activation and predicts clinical outcome segregated with TNM stage in gastric cancer patients following radical resection. *Journal of Translational Medicine*.

[194] Lamb J. J., Konda V. R., Quig D. W. (2011). A program consisting of a phytonutrient-rich medical food and an elimination diet ameliorated fibromyalgia symptoms and promoted toxic-element detoxification in a pilot trial. *Alternative Therapies in Health and Medicine*.

[195] Aydemir T. B., Blanchard R. K., Cousins R. J. (2006). Zinc supplementation of young men alters metallothionein, zinc transporter, and cytokine gene expression in leukocyte populations. *Proceedings of the National Academy of Sciences of the United States of America*.

[196] Mulder T. P. J., van der Sluys Veer A., Verspaget H. W. (1994). Effect of oral zinc supplementation on metallothionein and superoxide dismutase concentrations in patients with inflammatory bowel disease. *Journal of Gastroenterology and Hepatology*.

[197] Hu R., Hebbar V., Kim B. R. (2004). In vivo pharmacokinetics and regulation of gene expression profiles by isothiocyanate sulforaphane in the rat. *Journal of Pharmacology and Experimental Therapeutics*.

[198] Kimura T., Okumura F., Onodera A., Nakanishi T., Itoh N., Isobe M. (2011). Chromium (VI) inhibits mouse *metallothionein-I* gene transcription by modifying the transcription potential of the co-activator p300. *The Journal of Toxicological Sciences*.

[199] Weng C. J., Chen M. J., Yeh C. T., Yen G. C. (2011). Hepatoprotection of quercetin against oxidative stress by induction of metallothionein expression through activating MAPK and PI3K pathways and enhancing Nrf2 DNA-binding activity. *New Biotechnology*.

[200] Singh M., Tulsawani R., Koganti P., Chauhan A., Manickam M., Misra K. (2013). Cordyceps sinensis increases hypoxia tolerance by inducing heme oxygenase-1 and metallothionein via Nrf2 activation in human lung epithelial cells. *BioMed Research International*.

[201] Sales N. M. R., Pelegrini P. B., Goersch M. C. (2014). Nutrigenomics: definitions and advances of this new science. *Journal of Nutrition and Metabolism*.

[202] Lim U., Song M. A. (2012). Dietary and lifestyle factors of DNA methylation. *Methods in Molecular Biology*.

[203] Lang I. A., Galloway T. S., Scarlett A. (2008). Association of urinary bisphenol A concentration with medical disorders and laboratory abnormalities in adults. *The Journal of the American Medical Association*.

[204] Rezg R., El-Fazaa S., Gharbi N., Mornagui B. (2014). Bisphenol A and human chronic diseases: current evidences, possible mechanisms, and future perspectives. *Environment International*.

[205] Mostafalou S., Abdollahi M. (2013). Pesticides and human chronic diseases: evidences, mechanisms, and perspectives. *Toxicology and Applied Pharmacology*.

[206] Magliano D. J., Loh V. H. Y., Harding J. L., Botton J., Shaw J. E. (2014). Persistent organic pollutants and diabetes: a review of the epidemiological evidence. *Diabetes & Metabolism*.

[207] Agarwal S., Zaman T., Tuzcu E. M., Kapadia S. R. (2011). Heavy metals and cardiovascular disease: results from the National Health and Nutrition Examination Survey (NHANES) 1999–2006. *Angiology*.

[208] Rissman E. F., Adli M. (2014). Minireview: transgenerational epigenetic inheritance: focus on endocrine disrupting compounds. *Endocrinology*.

[209] Walker D. M., Gore A. C. (2011). Transgenerational neuroendocrine disruption of reproduction. *Nature Reviews Endocrinology*.

